# Multiple Comprehensive Analyses Identify Lysine Demethylase KDM as a Potential Therapeutic Target for Pancreatic Cancer

**DOI:** 10.7150/ijms.96134

**Published:** 2024-08-12

**Authors:** Wan-Jou Shen, Hsuan-Min Kao, Chih-Yang Wang, Rubina Kousar, Jing-Shan Lin, Ching-Chung Ko, Hung-Yun Lin, Hoang Dang Khoa Ta, Gangga Anuraga, Do Thi Minh Xuan, Sachin Kumar, Sanskriti Dey, Ngoc Phung Ly, Wei-Jan Wang

**Affiliations:** 1 Graduate Institute of Biomedical Sciences, College of Medicine, China Medical University, Taichung 40402, Taiwan.; 2 Department of Geriatric, Ditmanson Medical Foundation Chia-Yi Christian Hospital, Chiayi City 600566, Taiwan.; 3 Graduate Institute of Cancer Biology and Drug Discovery, College of Medical Science and Technology, Taipei Medical University, Taipei 11031, Taiwan; 4 Ph.D. Program for Cancer Molecular Biology and Drug Discovery, College of Medical Science and Technology, Taipei Medical University and Academia Sinica, Taipei 11031, Taiwan; 5 TMU Research Center of Cancer Translational Medicine, Taipei Medical University, Taipei 11031, Taiwan.; 6 Department of Biological Science and Technology, China Medical University, Taichung 40676, Taiwan; 7 Department of Medical Imaging, Chi-Mei Medical Center, Tainan, Taiwan; 8 Department of Health and Nutrition, Chia Nan University of Pharmacy and Science, Tainan, Taiwan; 9 School of Medicine, College of Medicine, National Sun Yat-Sen University, Kaohsiung, Taiwan.; 10 Cancer Center, Wan Fang Hospital, Taipei Medical University, Taipei 11031, Taiwan.; 11 Traditional Herbal Medicine Research Center of Taipei Medical University Hospital, Taipei Medical University, Taipei 11031, Taiwan.; 12 Pharmaceutical Research Institute, Albany College of Pharmacy and Health Sciences, Rensselaer, NY 12144, USA.; 13 Department of Statistics, Faculty of Science and Technology, Universitas PGRI Adi Buana, Surabaya, East Java 60234, Indonesia.; 14 Faculty of Pharmacy, Van Lang University, 69/68 Dang Thuy Tram Street, Ward 13, Binh Thanh District, Ho Chi Minh City 70000, Vietnam.; 15 Faculty of Biotechnology and Applied Sciences, Shoolini University of Biotechnology and Management Sciences, Himachal Pradesh, India.; 16 Natural Product Research Center, Korea Institute of Science and Technology (KIST), Gangneung 25451, Republic of Korea.; 17 Division of Bio-Medical Science and Technology, KIST School, University of Science and Technology (UST), Seoul 02792, Republic of Korea.; 18 Research Center for Cancer Biology, China Medical University, Taichung 40402, Taiwan.

## Abstract

Pancreatic cancer (PC) is a challenging and heterogeneous disease with a high mortality rate. Despite advancements in treatment, the prognosis for PC patients remains poor, with a high chance of disease recurrence. Biomarkers are crucial for diagnosing cancer, predicting patient prognosis and selecting treatments. However, the current lack of effective biomarkers for PC could contribute to the insufficiency of existing treatments. These findings underscore the urgent need to develop novel strategies to fight this disease. This study utilized multiple comprehensive bioinformatic analyses to identify potential therapeutic target genes in PC, focusing on histone lysine demethylases (KDMs). We found that high expression levels of KDM family genes, particularly KDM1A, KDM5A and KDM5B, were associated with improved overall survival in the cohort. Furthermore, the infiltration of various immune cells, including B cells, neutrophils, CD8^+^ T cells, dendritic cells, and macrophages, was positively correlated with KDM1A, KDM5A, and KDM5B expression. Moreover, MetaCore pathway analysis revealed interesting connections between KDM1A and the cell cycle and proliferation, between KDM5A and DNA damage and double-strand break repair through homologous recombination, and between KDM5B and WNT/β-catenin signaling. These findings suggest that KDM1A, KDM5A and KDM5B may serve as promising biomarkers and therapeutic targets for PC, a disease of high importance due to its aggressive nature and urgent need for novel biomarkers to improve diagnosis and treatment.

## Introduction

Pancreatic cancer (PC), a type of cancer that affects the digestive system, is associated with a 5-year survival rate of less than 5% and a median survival duration of 6 months in patients in whom the cancer cannot be surgically removed 1. Moreover, the majority of PCs are of the exocrine type, with ductal adenocarcinoma being the most common, accounting for 80-90% of all cases. 2. PC ranks as the 13^th^ most common type of cancer globally, with a total incidence of 458,000 cases, and is the seventh most lethal cancer type, accounting for approximately 50,000 deaths in the United States alone 3. The symptoms of PC may not be distinct, making early diagnosis challenging with current screening techniques 4. Therefore, understanding the molecular mechanisms that may induce treatment resistance, morbidity, and mortality may aid in devising relevant clinical strategies for treating PC 5.

Biomarkers are crucial in cancer diagnosis, prognosis prediction, and treatment selection. Samples of tissue, plasma, urine, and saliva can be analyzed to identify relevant biomarkers, providing a noninvasive, cost-effective, and efficient screening method 6. The development of novel biomarkers can improve early detection, prognosis prediction, and treatment selection, ultimately leading to better patient outcomes 7-11. Since PC is often diagnosed at an advanced stage when treatment options are limited, the discovery of new biomarkers could allow for earlier intervention and improved survival rates. Moreover, new biomarkers may also help monitor the effectiveness of treatments and develop more targeted therapies for this challenging disease 12-14.

Histone lysine demethylases (KDMs) have been demonstrated to remove methylation marks from lysine residues in histone tails, affecting gene transcription during development and cancer progression. Moreover, their activity can either promote or suppress tumor growth, depending on the cellular mechanism and specific isoforms of KDMs. The KDM family consists of 19 members: KDM1A, KDM1B, KDM2A, KDM2B, KDM3A, KDM3B, KDM4A, KDM4B, KDM4C, KDM4D, KDM4E, KDM5A, KDM5B, KDM5C, KDM5D, KDM6A, KDM6B, KDM7A, and KDM8 15. KDM1A is highly expressed in various human cancers, such as triple-negative breast cancer and cancer stem cells (CSCs). In prostate cancer, the overexpression of KDM1B is associated with poor prognosis. Inhibitors targeting KDM1B significantly reduce the development of prostate tumors 16. Additionally, in breast cancer, KDM2A is overexpressed and associated with poor prognosis 17. Similarly, in ovarian cancer, overexpression of KDM2B is associated with poor prognosis, whereas a decrease in KDM2B expression is correlated with reduced proliferation and migration of cancer cells 18. In lung cancer, the overexpression of KDM1A and KDM3A has been associated with lymphatic metastasis, pathological grade, and clinical stage. Another study reported that high KDM3B expression in breast cancer cells and tissues leads to rapid growth and invasion of cancer cells 19. Furthermore, KDM4A overexpression has been observed in malignant pleural mesothelioma, an aggressive malignancy with a poor prognosis 20. Moreover, a study indicated that KDM4C expression substantially increased in gastric CSCs and correlated favorably with relevant biomarkers 21. In addition, KDM4D promotes leukemia progression by activating the expression of myeloid leukemia proteins 22. Although evidence suggests that the KDM family plays a crucial role in cancers, an overview of KDMs in PC is still lacking.

**Figure 1:**
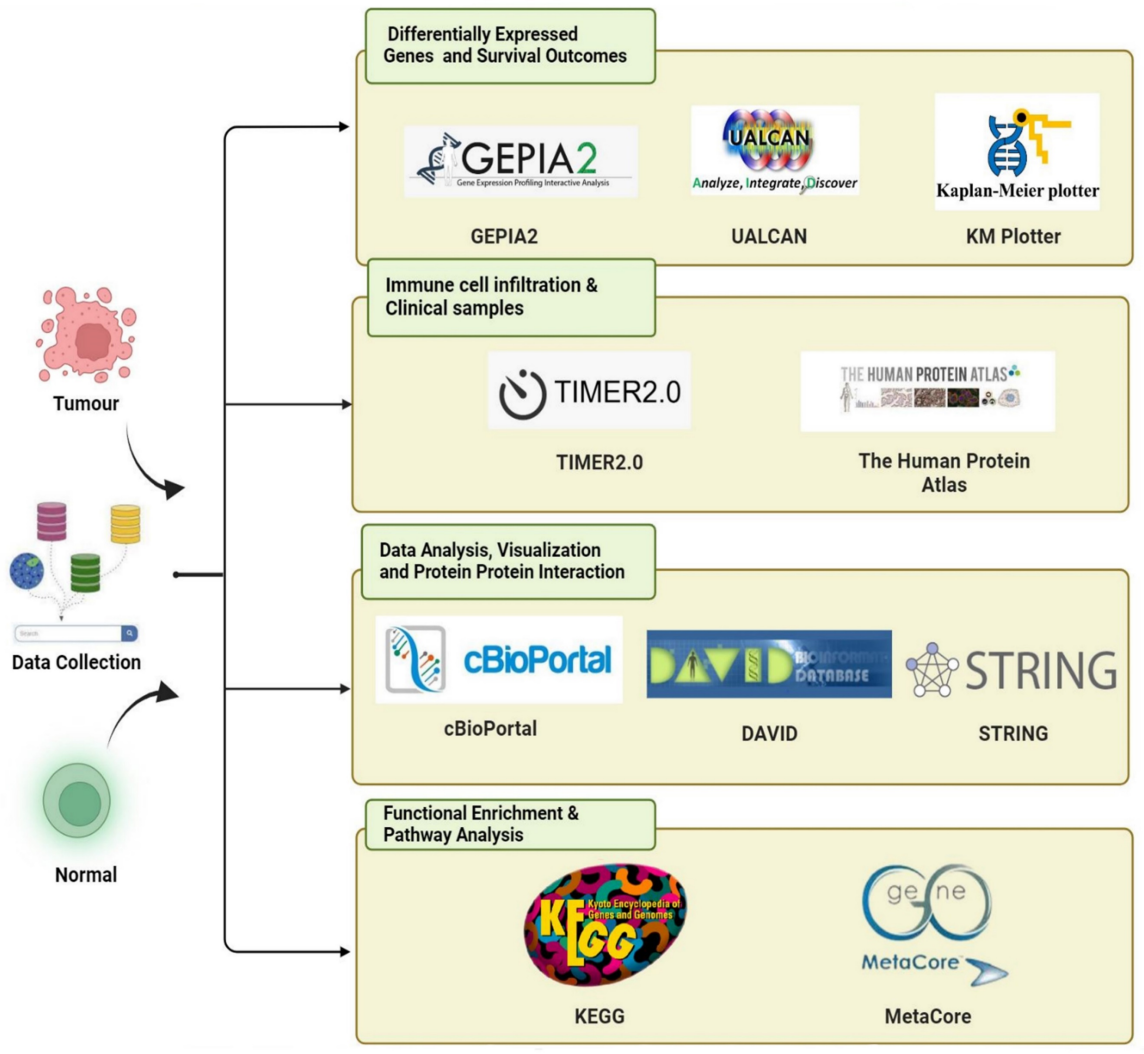
The bioinformatics analysis flowchart depicts the processing of publicly available data from TCGA and GTEx databases.

In this study, we investigated the overexpression patterns of different members of the KDM family in PC and focused on all KDM family members through comprehensive bioinformatics analysis (**Fig. 1**.). To understand the importance of the KDM family in PC development and prognosis, we analyzed relevant data from the Genotype-Tissue Expression databases and The Cancer Genome Atlas (TCGA) 23. The differential expression levels of various KDM family genes in PC were observed via data obtained from the Gene Expression Profiling Interactive Analysis 2 (GEPIA2) and University of Alabama at Birmingham CANcer (UALCAN) databases. This observation indicated their potential oncogenic and tumor-suppressive activities in PC. We further evaluated the diagnostic and prognostic value of KDM family members and assessed their protein expression levels in PC. Overall, we found that KDM1A, KDM5A, and KDM5B were highly expressed and critical for prognostic assessment and immune infiltration in PC. These findings enhance our understanding of the role of KDMs in PC and provide novel biomarkers for PC treatment, enabling the prediction of prognosis on the basis of KDM functions.

## Materials and Methods

### GEPIA2

We collected data on the expression of hub genes in both normal and malignant tissues from the GEPIA2 database (http://gepia2.cancer-pku.cn/index.html). We then investigated the differences in hub gene expression to validate the data obtained from TCGA. Additionally, we analyzed the expression patterns of the target genes in various cancers and the correlations between target gene expression and tumor grade via data from the GEPIA2 database 23-26.

### UALCAN

The UALCAN online portal (http://ualcan.path.uab.edu) provides data on patient survival, cancer transcriptomes, and proteomics. Using data from TCGA, UALCAN users can analyze the expression of protein-coding genes and their impact on patient survival across 33 types of cancers 27-29. In this study, we examined the expression profiles of all 19 members of the KDM family in patients with PC.

### Survival analysis

The KM survival curve estimates the likelihood of patient survival over time by dividing time into short intervals. We utilized the KM plotter (http://kmplot.com/analysis/) tool to assess the predictive value of the mRNA expression levels of various KDM family genes in PC. Patients with PC were classified into high- and low-expression groups on the basis of the median values of mRNA expression, and their overall survival (OS) was evaluated. The KM database provides data on hazard ratios, 95% confidence intervals, median mRNA expression, and p values, making it a valuable resource for evidence-based survival data. Additionally, we determined the log-rank p values and hazard ratios (HRs) with 95% confidence intervals 30-32.

### Tumor Immune Estimation Resource

The Tumor Immune Estimation Resource (TIMER: http://timer.comp-genomics.org) online server is a comprehensive tool for systematically assessing infiltrating immune cells in different cancers. We utilized this tool to estimate the abundance of six types of immune cells: CD4^+^ T cells, neutrophils, B cells, dendritic cells, CD8^+^ T cells, and macrophages. Using the TIMER database, we examined the correlations between KDM1A, KDM5A, and KDM5B expression and the infiltration of various immune cells. TCGA data were analyzed via TIMER to assess KDM1A, KDM5A, and KDM5B expression patterns in both PC and normal tissues 33-35. The gene symbols for KDM1A, KDM5A, and KDM5B are plotted on the x-axis, whereas the related marker genes' symbols are plotted on the y-axis. Gene expression levels are represented as log2 RSEM values.

### Protein-protein interaction network and gene coexpression

We utilized the Search Tool for the Retrieval of Interacting Genes/Proteins (STRING: https://string-db.org/cgi/input) database to investigate protein‒protein interactions (PPIs). KDM1A, KDM5A, and KDM5B sequences were queried in the STRING database to identify their PPIs. On the basis of the overexpression patterns of these proteins, we constructed a PPI network 36-38.

### Functional component and potential pathway analyses

We employed cBioPortal for Cancer Genomics (https://www.cbioportal.org) software to analyze the coexpression of selected genes with other genes. The relevant data were subsequently uploaded to the Database for Annotation, Visualization, and Integrated Discovery (DAVID: https://david.ncifcrf.gov), a tool for systematically gathering biological data from multiple genes 39-41, and an online platform for data analysis and visualization (http://www.bioinformatics.com.cn/). Additionally, we obtained data from the Kyoto Encyclopedia of Genes and Genomes (KEGG), which assigns biological functions to genes and genomes, whereas DAVID provides information about gene products, processes, and functions. To gain a deeper understanding of the biological processes associated with the differentially expressed genes (DEGs), we employed a curated pathway enrichment analysis using MetaCore™ (Clarivate Analytics, UK) (https://clarivate.com/products/biopharma/research-development/early-research-intelligence-solutions/). For each time point, we uploaded the DEGs relative to control cells into MetaCore™ for pathway analysis. The Pathway Maps tool was then used to identify significantly enriched pathways associated with these DEGs. An adjusted p value threshold of less than 0.05 was applied for the analysis, and all expression changes were included. The results are presented as -log(p value) values, ranked by their statistical significance 42-44.

### Immunohistochemical (IHC) analysis

PC tissue microarrays, sourced from TissueArray (PA2082a), were analyzed to explore the expression of KDM1A, KDM5A, and KDM5B via IHC staining. The microarrays included normal and tumor samples from PC patients 45-47. For the IHC staining, we used antibodies against KDM1A (Abcam, ab37165), KDM5A (Abcam, ab92533), and KDM5B (Abcam, ab244220). The staining procedures were performed according to the TnAlink Polymer Detection System (DAB) manufacturers' protocols (BioTnA, TAHC04D).

### Statistical analysis

We conducted survival analysis via the KM plotter tool with its default settings. Recurrence-free survival was the primary focus, utilizing the automatically calculated best cutoff values and the J best probe set. Furthermore, we utilized the TCGA Pan-Cancer Atlas dataset from the cBioPortal database to acquire patient data and examine the impact of the expression of various KDM family genes on overall survival (OS). We determined all potential cutoff values between the lower and upper quartiles, selecting the best-presenting threshold as the final cutoff value. A log-rank p value less than 0.05 was considered statistically significant 48-50.

## Results

### Differential expression analysis of the KDM family genes in PC

**Figure 2:**
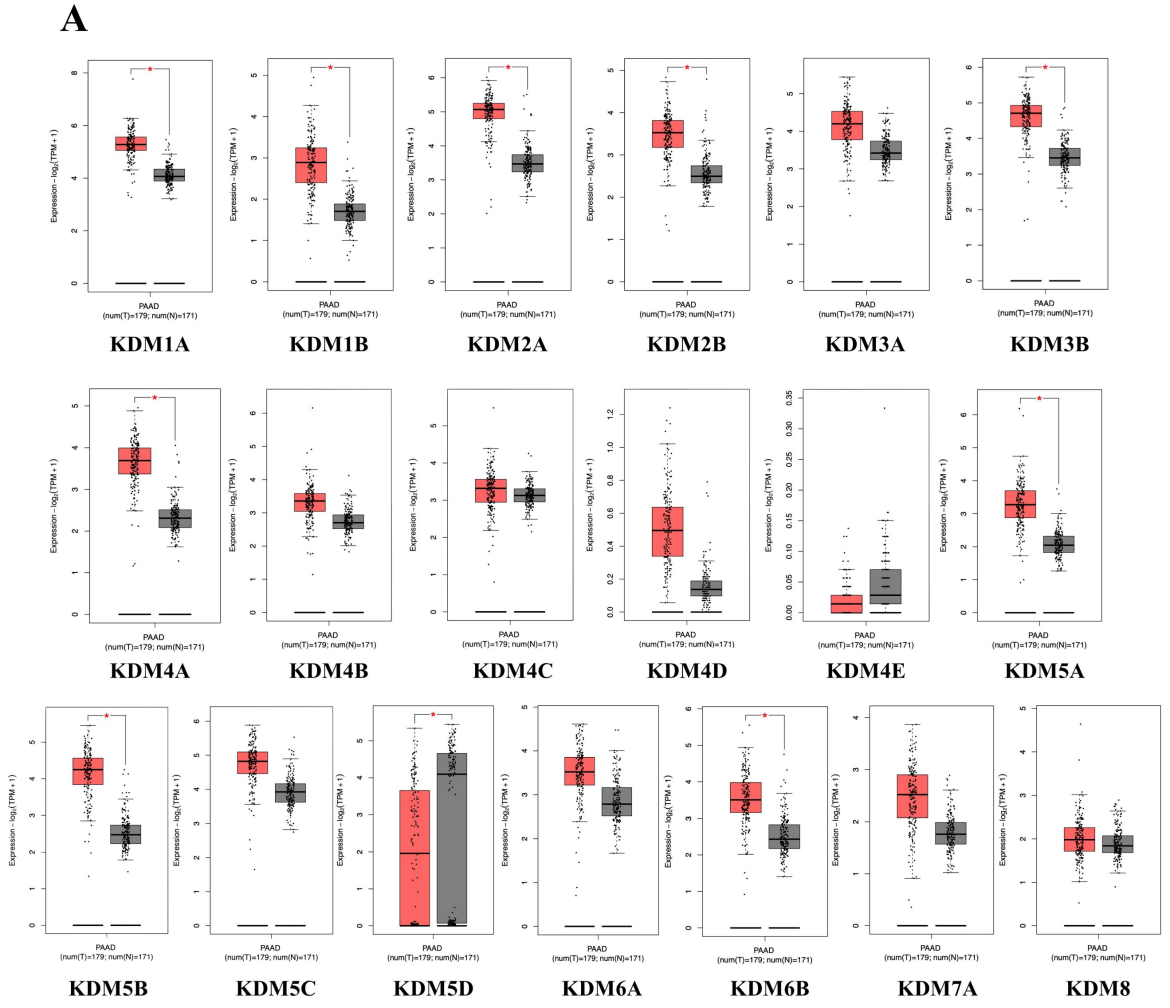
** The expression levels of KDM family proteins in pancreatic cancer (PC).** KDM family mRNA expression analysis in PCs from the GEPIA2 database. Box plot (GEPIA2): normal tissue, gray; tumor tissue, red. *, p<0.01.

KDMs are critical enzymes that regulate gene expression by removing methyl groups from lysine residues on histones. This process is essential for maintaining the dynamic balance of genomic expression, ensuring genome integrity, and supporting epigenetic inheritance. Aberrant KDM expression and activity in cancer are pivotal in tumor progression and drug resistance. However, no study has explored the overall role and expression of the entire KDM family in PC. This study utilized an extensive range of publicly available databases and web-based analysis tools. The bioinformatics method we employed is depicted in **Figure 1**. To identify the role of KDMs in PC, we investigated the expression patterns of various KDM family genes in both normal and cancerous tissues from PC patients via the GEPIA2 database. Our findings revealed significantly upregulated expression levels (p < 0.5) of KDM1A, KDM1B, KDM2A, KDM2B, KDM3B, KDM4A, KDM5A, KDM5B, and KDM6B in PC tissue, suggesting their potential oncogenic role** (Fig. 2)**. However, KDM3A, KDM4B, KDM4C, KDM4D, KDM4E, KDM5C, KDM6A, KDM7A, and KDM8 were not significantly different in the tumor tissue. Furthermore, immunohistochemical data analysis of the expression of KDM5B in human tissue, which is available in the Human Protein Atlas database, revealed greater staining intensity in PC tissue than in normal tissue. These findings suggest that certain members of the KDM family could serve as promising biomarkers for PC.

### The prognostic relevance of KDM family gene expression in PC and its correlation with the infiltration of immune cells

**Figure 3:**
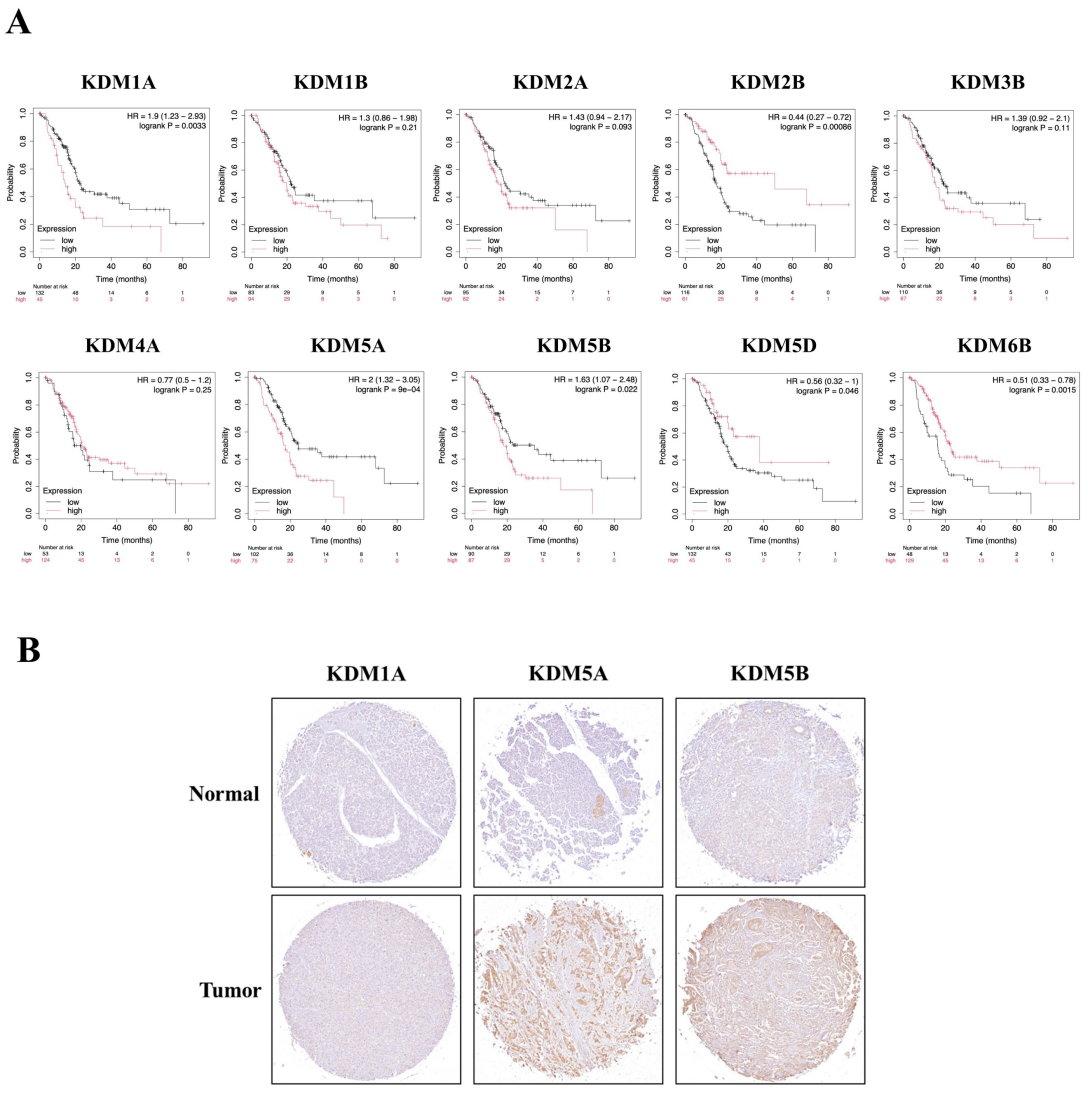
** The prognostic value of the mRNA levels of the KDM family in patients with pancreatic cancer (PC) was determined via Kaplan‒Meier plotter analysis.** (A) The hazard ratio (HR) indicates the prognostic value for PC patients. The log [rank p] test was used to determine the level of prognostic significance, with a value of p<0.05 considered significant. High expression of KDM1A/5A/5B was significantly associated with poor prognosis, indicating poorer outcomes. Conversely, the HRs of KDM2B/5D/6B were significantly lower, suggesting better prognostic outcomes in patients with pancreatic cancer. (B) Immunohistochemical patterns of KDM1A, KDM5A, and KDM5B expression in normal and tumor tissues from patients with PC.

**Figure 4:**
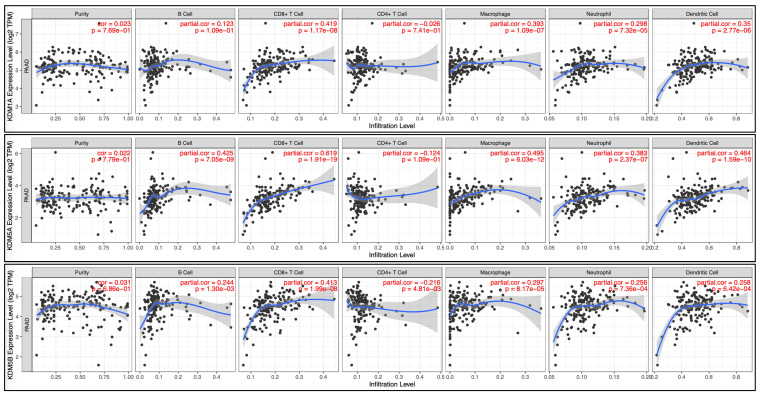
** Correlation analysis was performed to assess the relationships between differentially expressed KDM family genes and immune cell infiltration via the TIMER database.** The figure illustrates the associations of KDM1A, KDM5A, and KDM5B gene expression with tumor purity and markers of tumor-infiltrating immune cells, including B cells, CD8^+^ T cells, CD4^+^ T cells, macrophages, neutrophils, and dendritic cells. Spearman correlations were used to assess the relationships between these KDM genes and the mentioned immune cells, with statistical significance set at p<0.05.

We then examined whether the prognosis of cancer patients was related to the overexpression of KDM1A, KDM1B, KDM2A, KDM2B, KDM3B, KDM4A, KDM5A, KDM5B, KDM5D, and KDM6B in PC tumor tissue. The results revealed that high expression levels of KDM family genes were associated with two opposite scenarios in PC patients: the high expression levels were linked to either extended or poorer overall survival (OS) in patients with PC. Notably, poor overall survival (OS) was observed for KDM1A, KDM5A, and KDM5B (p < 0.05). Conversely, KDM2B, KDM4B, KDM4C, KDM4D, KDM4E, KDM5D, KDM6B, and KDM8 were associated with prolonged OS **(Fig. 3A)**. Furthermore, to assess the protein expression levels of KDM1A, KDM5A, and KDM5B in human tumor tissue, we conducted IHC on a tumor tissue microarray. In clinical PC samples, significantly greater expression of KDM1A, KDM5A, and KDM5B was observed than in normal pancreatic tissue **(Fig. 3B)**. These results suggest that the KDM family, particularly KDM1A, KDM5A, and KDM5B, plays an oncogenic role and is associated with poor prognosis in PC patients. In addition, accumulating studies have demonstrated that immune infiltration in PC is crucial in the development of more effective immunotherapies, as it influences disease progression, treatment response, and patient outcomes 51. Therefore, we were interested in investigating whether KDM1A, KDM5A, and KDM5B expression was correlated with the enrichment of tumor-infiltrating lymphocytes in PC. Using the TIMER database, we found that the expression of the KDM1A gene is associated with the infiltration of CD8^+^ T cells, macrophages, neutrophils, and dendritic cells. Furthermore, KDM5A was related to the infiltration of B cells, CD8^+^ T cells, macrophages, neutrophils, and dendritic cells. Additionally, KDM5B was associated with the infiltration of B cells, CD8^+^ T cells, CD4^+^ T cells, macrophages, neutrophils, and dendritic cells in patients with PC **(Fig. 4)**. These results suggest that KDM1A, KDM5A, and KDM5B may serve as oncogenic biomarkers and correlate with antitumor immunity in PC.

### Analysis of gene interactions and molecular functions involving KDM1A, KDM5A, and KDM5B

**Figure 5:**
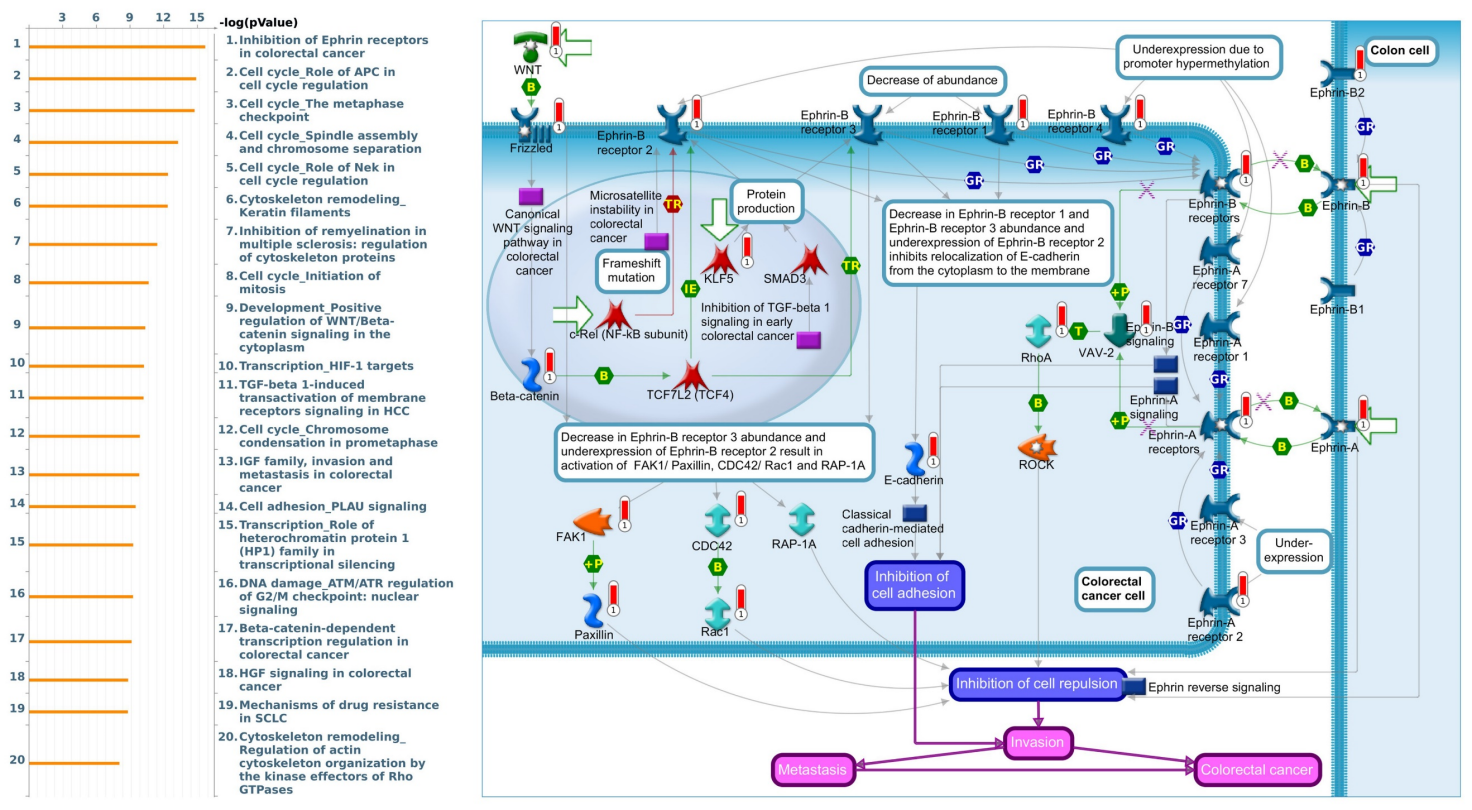
** MetaCore pathway analysis of the coexpression gene network involving KDM1A in pancreatic cancer.** We extracted the top 1000 genes coexpressed with KDM1A from the TCGA database. We conducted a pathway analysis and generated a pathway list ordered by the -log p value. The "Ephrin receptors pathway" ranked highest in the biological process category. The figure illustrates interactions between genes and proteins, with symbols representing proteins and arrows depicting protein interactions (green for activation, red for inhibition).

**Figure 6:**
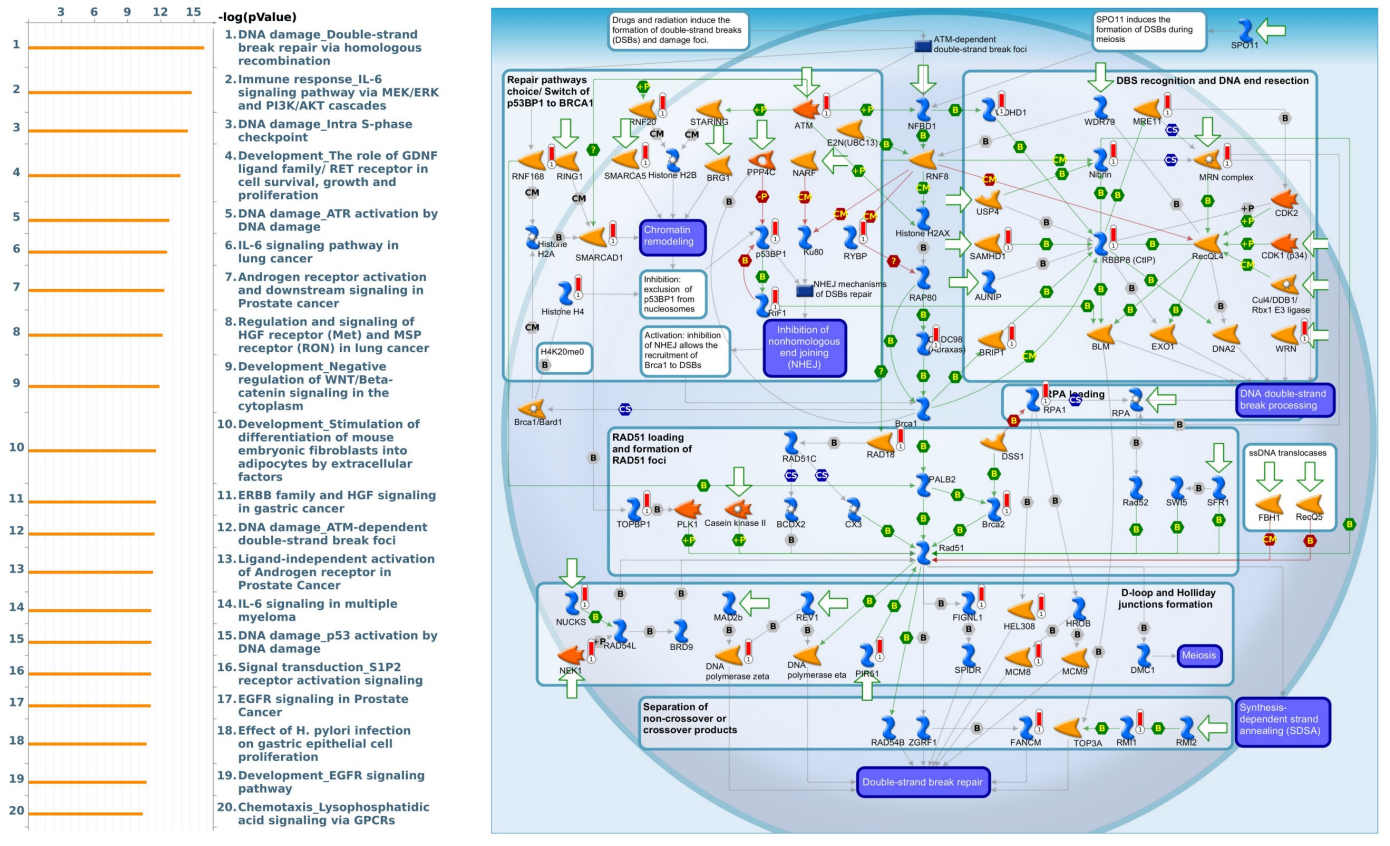
** Expression of the KDM5A signaling pathway in pancreatic cancer (MetaCore).** We used the MetaCore platform to analyze genes coexpressed with KDM5A from the associated TCGA database. Our analysis revealed that "DNA damage" was the most common biological process.

**Figure 7:**
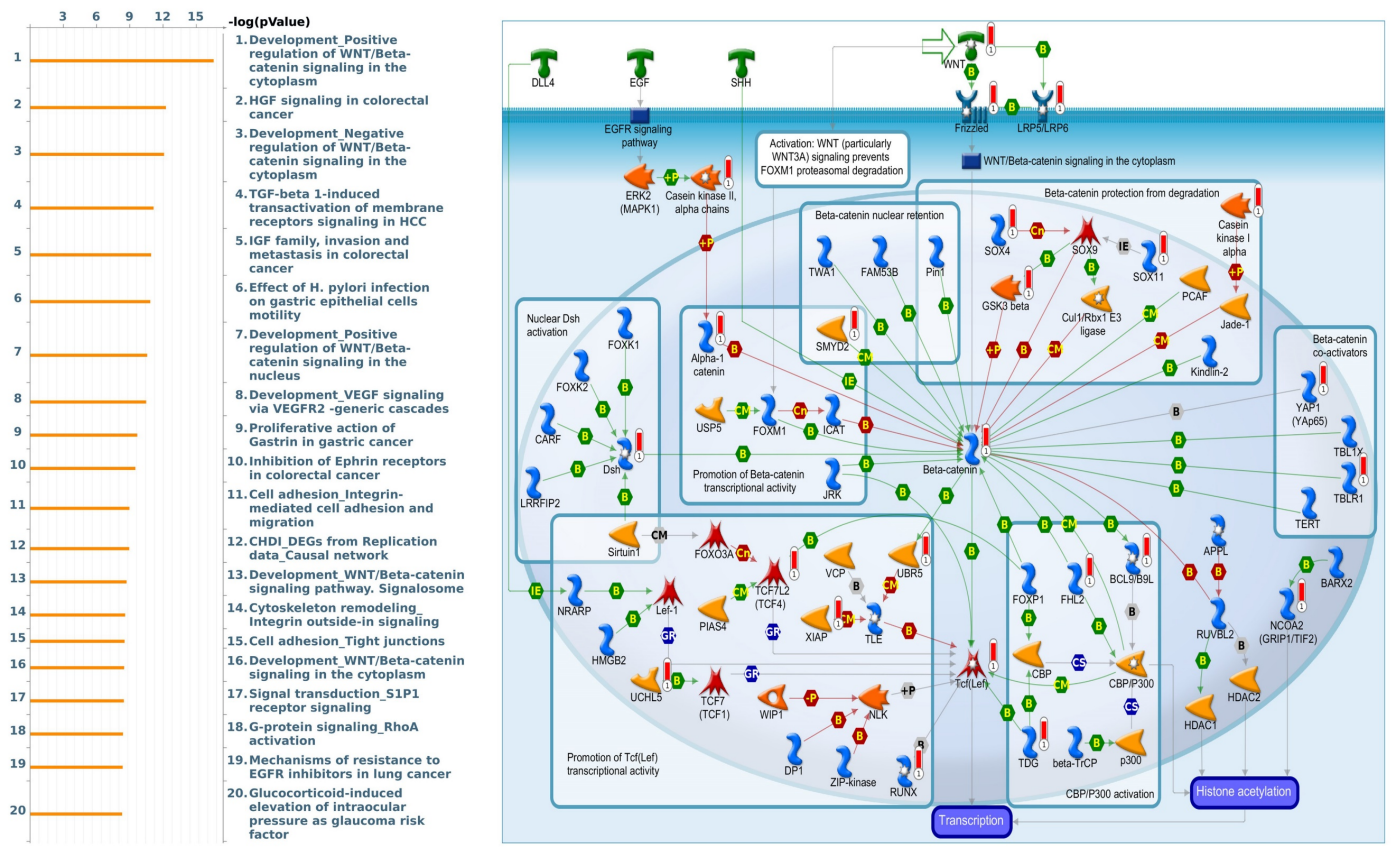
** MetaCore enrichment pathway analysis of genes coexpressed with KDM5B in pancreatic cancer.** We used the MetaCore platform to analyze genes coexpressed with KDM5B from the associated TCGA database. Our analysis revealed that "WNT/β-catenin signaling" ranked highest in the biological process analysis.

To gain further insight into the molecular regulation of KDMs, we used the STRING database to identify PPIs within the KDM family. The results indicated that KDM1A and KDM5A significantly interact with HDAC1/2, which plays a critical role in cancer by regulating gene expression through histone deacetylation, thereby influencing cell proliferation, apoptosis, and metastasis 52. Moreover, we found that KDM5B interacts with HDAC1/4 to mediate miRNA expression and enhance docetaxel resistance in lung adenocarcinoma **(Supplementary Fig. 1A)** 53. Furthermore, we utilized gene sets coexpressed with KDM1A, KDM5A, and KDM5B from the TCGA database. Our analysis revealed several pathways associated with KDM1A that are significantly associated with pancreatic cancer, including ephrin receptor signaling, cell cycle regulation, carbon metabolism, progesterone-mediated oocyte maturation, and glycolysis/glycogenesis, as identified by MetaCore analysis and KEGG database **(Fig. 5 and Supplementary Fig. 1B)**. Our findings provide additional insights into the functional roles of KDM5A, revealing its association with pathways related to DNA damage, the IL-6-mediated immune response, the DNA damage response, and double-strand break repair through homologous recombination **(Fig. 6 and Supplementary Fig. 1B)**. Moreover, our study demonstrated that KDM5B is associated with WNT/β-catenin signaling in the cytoplasm, HGF signaling in cancer, sphingolipid metabolism, and the Hippo pathway **(Fig. 7 and Supplementary Fig. 1B)**. Furthermore, we identified the top 50 pathways related to KDM1A, KDM5A and KDM5B, and the results revealed that the KDM family may contribute to multiple oncogenic pathways in PC **(Supplementary Tables 1-3)**. These correlations offer additional evidence of their potential participation in crucial cellular processes and signaling pathways relevant to cancer biology. Taken together, in this study, we conducted a comprehensive analysis via multiple credible databases, which implied that KDMs, particularly KDM1A, 5A, and 5B, may play critical roles in the molecular pathways underlying the development and progression of PC **(Fig. 7)**.

## Discussion

While PC has a lower incidence than other malignancies, such as breast, lung, colorectal, and prostate cancers, it was the third leading cause of cancer-related death in 2021 54. By 2030, it is projected to become the second leading cause of cancer-related death in the United States 55. Despite this, there has been limited success in improving the outcomes of PC over the past two decades, underscoring the need for effective treatment and diagnostic approaches.

Although key genetic mutations, such as those in KRAS, TP53, SMAD4, and CDKN2A, have been identified in PC patients, the overall outcomes for these patients have not been markedly improved. Therefore, identifying novel biomarkers for PC treatment is imperative as the key to improving patient outcomes and extending survival. By meticulously analyzing these biomarkers and their correlation with treatment response, researchers can pave the way for more effective therapeutic strategies. This holds particular promise for KDMs, enzymes that regulate gene expression through histone methylation, offering hope for improved patient care and increased survival rates. Although their involvement in cancer is well known, the molecular functions, expression patterns, and significance of KDM family genes in patient survival in PC are still unclear. In this study, we used multiple comprehensive bioinformatics approaches to analyze KDM family proteins in PC. Our findings suggest that KDM1A, KDM5A, and KDM5B could serve as biomarkers for PC and may be targeted to improve long-term patient survival.

We found that KDM1A, also known as LSD1, is overexpressed in PC and plays a significant role in disease development and progression, which is also correlated with the glycolysis pathway. Several KDM genes, including KDM1A, KDM5A, and KDM5B, were upregulated in PC tissues compared with normal controls, indicating their potential as oncogenes. Furthermore, high KDM1A, KDM5A and KDM5B expression correlated with poorer overall survival in PC patients. These results highlight these KDM family members as potential diagnostic and prognostic markers for PC. Studies have demonstrated that glycolysis is a critical regulator in cancer and plays a role in cancer stemness and chemoresistance 56. Chemotherapy is known to trigger cancer stemness, which is characterized by enhanced DNA repair capacity and contributes to temozolomide (TMZ) resistance in glioblastoma (GBM). High expression of KDM1A has been linked to therapy resistance and recurrence in GBM. Inhibiting KDM1A has been shown to sensitize glioma stem cells (GSCs) to TMZ, indicating its involvement in TMZ resistance 57. Furthermore, KDM1A acts as a coregulator for hormone receptors (AR, ER, GR, MR) and may control ERβ expression in ovarian cancer (OCa). KDM1A knockdown enhances ERβ expression, and combination therapy with a KDM1A inhibitor and ERβ agonist reduces OCa cell viability and invasion and promotes apoptosis in various OCa models 58.

In PC, inducing KDM2B protein expression has been shown to promote tumor progression through interaction with Polycomb group proteins to repress developmental genes. Furthermore, researchers have demonstrated that KDM2B collaborates with KDM5A and that Myc activates genes involved in ribosomal and mitochondrial functions, which are crucial for the metabolism and growth of PC​ 59​. Our study revealed that KDM2B gene expression is correlated with favorable prognosis in patients with PC. Therefore, gene expression and protein expression may be regulated by different mechanisms in PC, which requires further investigation. Intriguingly, our data revealed a link between KDM expression and immune cell infiltration in PC. Specifically, KDM1A, KDM5A, and KDM5B expression correlated with the infiltration of multiple immune cell types, including CD8^+^ T cells, B cells, macrophages and dendritic cells. These findings suggest that targeting these KDM genes may target cancer cells directly and modulate the antitumor immune response. Further investigations are needed to explore this possibility and its potential for immunotherapeutic strategies in PC. These enzymes play significant roles in a wide range of physiological and pathological processes, indicating their complex and context-dependent functions. Moreover, our results revealed that KDM5A and KDM5B are correlated with DNA damage repair and the WNT/β-catenin signaling pathway. Consistently, recent studies have shown that KDM5A can be upregulated in tumor tissues compared with normal prostate tissues and may promote malignancy through the regulation of chromosome instability 60, 61. Furthermore, research has demonstrated that KDM5B regulates the androgen receptor and interacts with the PI3K/AKT pathway, contributing to the progression of prostate cancer 62. In addition to KDM2B, we also found that KDM5D and KDM6B are correlated with good prognosis in PC patients. According to a previous study, KDM5D plays a critical role in prostate cancer by regulating the response to androgen deprivation therapy (ADT) and taxane treatments. Loss of KDM5D increases the expression of MYBL2, a transcription factor for which elevated levels are associated with poorer clinical outcomes, leading to resistance to ADT and taxanes 63. However, the role of KDM5D in PC remains unclear. In addition, KDM6B acts as a tumor suppressor in PC by demethylating histone H3 lysine 27, thereby controlling the expression of CEBPA, a key gene associated with cancer progression. Inhibition of KDM6B expression enhances PC cell aggressiveness, increases metastasis, and disrupts cellular senescence mediated by oncogenic KRAS, highlighting its critical role in maintaining cellular control in early cancer stages 64. Moreover, KDM6B has also been demonstrated to promote neuroblastoma cell differentiation by removing the repressive chromatin marker H3K27me3. KDM6B expression is induced by retinoic acid via HOXC9 and inhibits tumor cell growth by promoting cell differentiation 65. These findings highlight the complex roles of KDMs in cancer biology, particularly in PC, where they can influence both the suppression and activation of different pathways critical for cancer progression.

In addition, previous studies have demonstrated that KDM5A and KDM5C are overexpressed in gemcitabine-resistant pancreatic cancer cells and that their expression is regulated by CD44, a stem cell marker linked to chemoresistance. Moreover, studies have shown that KDM5 family members are involved in several critical oncogenic pathways, including epithelial‒mesenchymal transition (EMT) 66. These findings suggest that members of the KDM family could serve as new biomarkers or potential therapeutic targets for overcoming drug resistance in PC. In our study, MetaCore analysis suggested that KDM5A may contribute to the regulation of the IL-6 pathway. Consistent with this finding, KDM5A has been demonstrated to regulate osteosarcoma cell viability through the IL-6/JAK/STAT3 pathway, highlighting its potential role in modulating this signaling axis 67. Moreover, a previous study showed that KDM5B, by interacting with TIEG1, enhances the repression of SMAD7 thereby augmenting TGF-β signaling and potentially suppressing tumorigenesis 68. Interestingly, our bioinformatics analysis revealed that KDM5B is involved in multiple pathways, such as the WNT, HGF, and TGF-β pathways, in PC. Taken together, these findings showed that both KDM5A and KDM5B might act as protumorigenic factors in cancer, highlighting their potential as targets for therapeutic intervention. However, further research is needed to fully elucidate the molecular mechanisms underlying their roles, especially in PC progression, and to explore their potential as therapeutic targets in PC treatment.

## Conclusions

We conducted comprehensive bioinformatics analyses using data from the TCGA and the Genotype-Tissue Expression databases. We observed high expression levels of KDM family genes in PC tissue. Our prognostic analysis suggested that KDM1A, KDM5A, and KDM5B could be promising biomarkers and therapeutic targets for PC. In addition, we discovered that the elevated expression of KDM1A, KDM5A, and KDM5B in PC may be correlated with poor prognosis and significantly decreased immune cell infiltration. These data suggest that targeting KDMs could increase the effectiveness of immunotherapy for PC.

## Supplementary Material

Supplementary figure and tables.
