# Supplementary material for: Multiple Comprehensive Analyses Identify Lysine Demethylase KDM as a Potential Therapeutic Target for Pancreatic Cancer
Source: Int J Med Sci. 2024 Aug 12;21(11):2158–69. doi: 10.7150/ijms.96134 (PMC11373554; doi:10.7150/ijms.96134)
Supplement: Supplementary file 1 — Supplementary figure and tables. [file ijmsv21p2158s1.pdf]

Supplementary Figure 1. Protein–protein interaction and molecular pathway analysis of KDM1A, KDM5A, and KDM5B in pancreatic cancer. (A) Protein–protein interactions (PPIs) of KDM1A/5A/5B (STRING database). Highly interacting proteins are represented as hub protein nodes in the PPI network. (B) Molecular pathway analysis of coexpressed KDM1A/5A/5B genes was conducted via the KEGG database.

Supplementary Figure 1

A

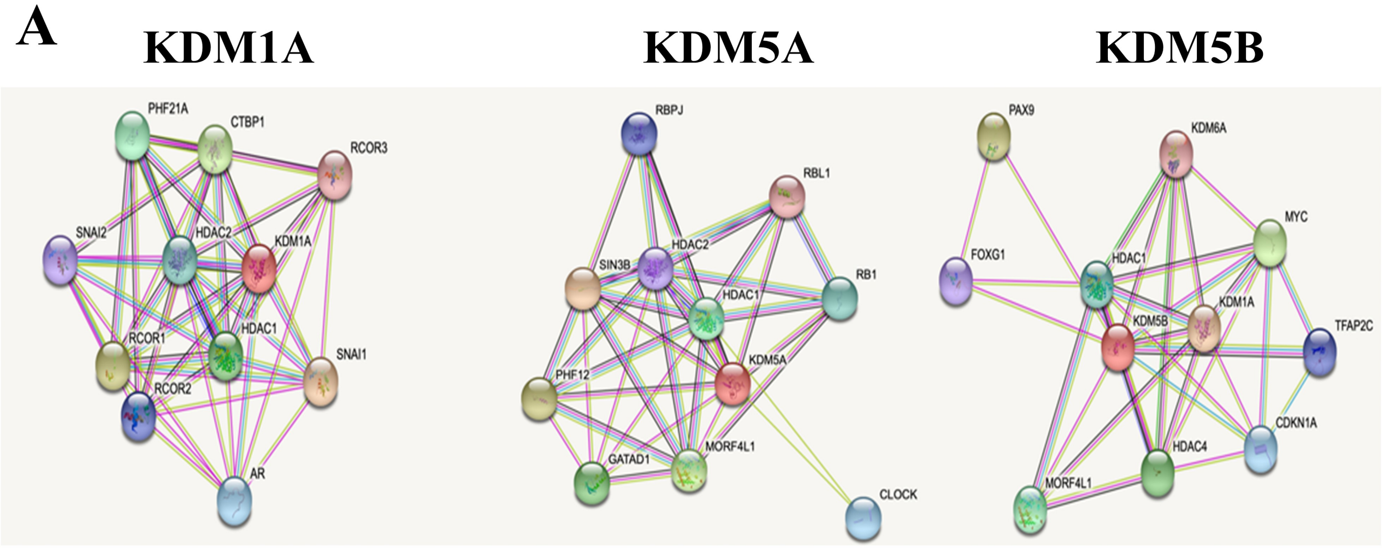

B

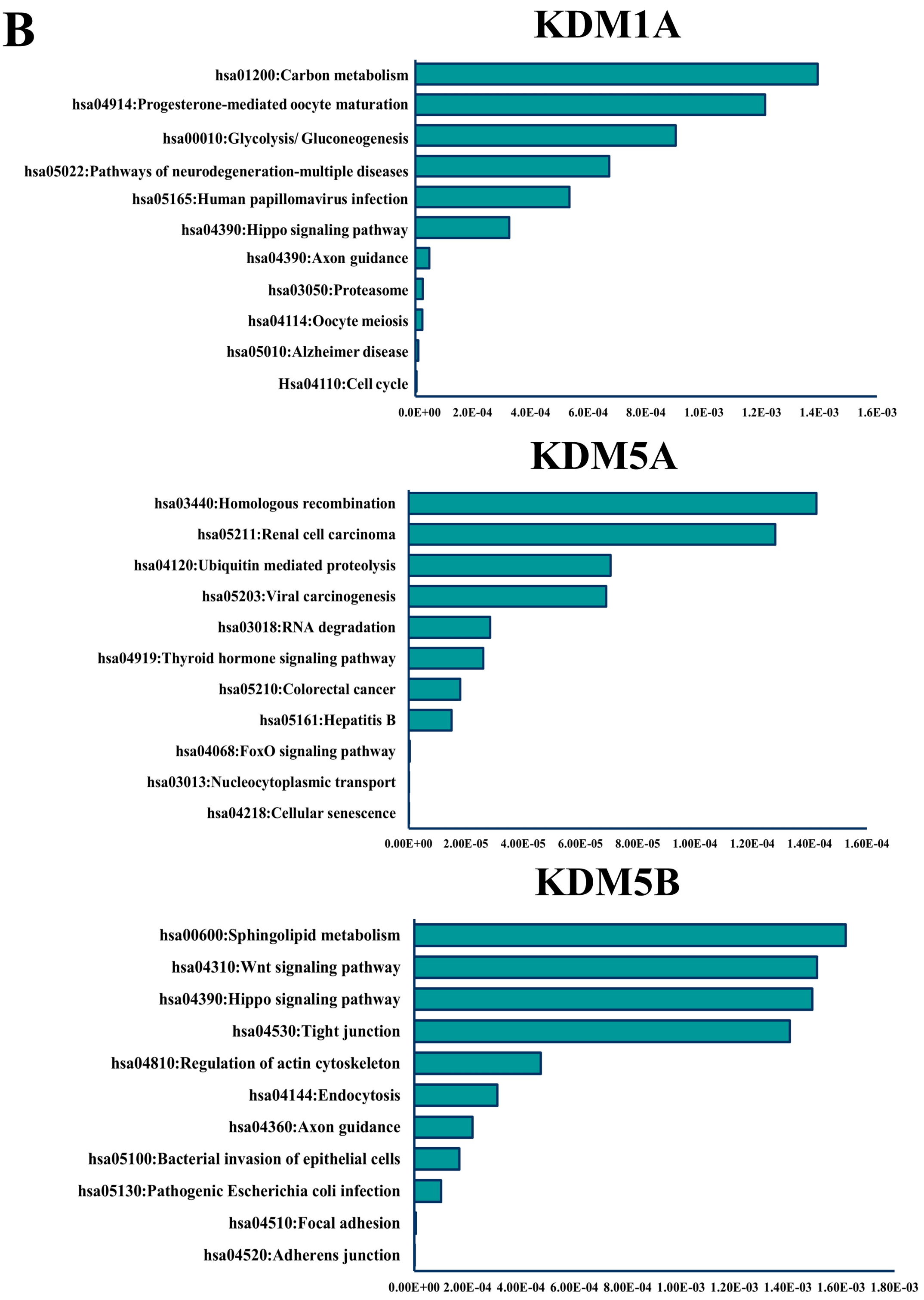

Supplementary Table S1: Pathway analysis of genes coexpressed KDM1A from TCGA pancreatic adenocarcinoma databases using the MetaCore database (with p<0.05 set as the cutoff value).

#	Maps	p-value	Network Objects from Active Data
**1**	Inhibition of Ephrin receptors in colorectal cancer	2.03E-16	RhoA, E-cadherin, Ephrin-B receptor 4, KLF5, Ephrin-B, WNT, Ephrin-B receptor 2, VAV-2, FAK1, Beta-catenin, CDC42, Ephrin-A receptors, Ephrin-B receptor 1, Ephrin-A, Ephrin-B2, Ephrin-B receptors, Ephrin-A receptor 2, Frizzled, Paxillin, Rac1
**2**	Cell cycle_Role of APC in cell cycle regulation	1.33E-15	CDC18L (CDC6), Cyclin B, ORC1L, CKS1, Nek2A, CDC20, MAD2a, BUBR1, MAD2b, CDH1, Tome-1, Cyclin A, Kid, CDC25A, BUB1, Aurora-A, PLK1, Securin, CDK1 (p34), CDK2
**3**	Cell cycle_The metaphase checkpoint	1.88E-15	Dynein 1, cytoplasmic, heavy chain, Survivin, Nek2A, CENP-A, SPBC24, CDC20, MAD2a, CENP-H, BUBR1, MAD2b, SPBC25, Rod, DSN1, HEC, CENP-E, BUB1, Aurora-A, PLK1, HP1 gamma, CENP-F, Zwilch
**4**	Cell cycle_Spindle assembly and chromosome separation	5.88E-14	Tubulin alpha, Cyclin B, Dynein 1, cytoplasmic, heavy chain, Nek2A, CDC20, MAD2a, Importin (karyopherin)-alpha, RCC1, KNSL1, HEC, Kid, TPX2, CSE1L, Dynein 1, cytoplasmic, intermediate chains, Aurora-A, Securin, Ran, CDK1 (p34), Tubulin (in microtubules)
**5**	Cell cycle_Role of Nek in cell cycle regulation	4.76E-13	Tubulin beta, PI3K cat class IA, Tubulin alpha, Histone H3, Nek2A, IRS-1, MAD2a, RCC1, HEC, Cyclin B1, TPX2, PDK (PDPK1), Nek11, Aurora-A, Ran, Histone H1, CDK1 (p34), Tubulin (in microtubules)
**6**	Cytoskeleton remodeling_Keratin filaments	5.00E-13	Plectin 1, Tubulin beta, Keratin 17, Keratin 5/14, Keratin 4, PPL(periplakin), Keratin 7, Keratin 13, Tubulin alpha, Keratin 14, Keratin 19, Keratin 5, Keratin 16, Keratin 6C, Keratin 6A, Keratin 4/13, Plakophilin 1, CDK1 (p34), Tubulin (in microtubules)

**7**	Inhibition of remyelination in multiple sclerosis: regulation of cytoskeleton proteins	4.46E-12	MLCP (cat), Tubulin beta, RhoA, Tubulin alpha, alpha-V/beta-1 integrin, MRLC, Stathmin, Tubulin beta 4, p190RhoGAP, FAK1, chTOG, Myosin II, CDC42, TGF-beta, Sirtuin2, MELC, Fibronectin, Paxillin, Rac1, Tubulin (in microtubules)
**8**	Cell cycle_Initiation of mitosis	2.65E-11	Wee1, Cyclin B2, Histone H3, CDC25C, FOXM1, Lamin B, KNSL1, p90RSK1, AKT(PKB), AP-2A, Cyclin B1, PLK1, MAT1, Histone H1, CDK1 (p34)
**9**	Development_Positive regulation of WNT/Beta-catenin signaling in the cytoplasm	5.31E-11	Casein kinase II, alpha chains, COX-2 (PTGS2), ITGB1, SIAH2, IRS-2, SIAH1, IRS-1, WNT, PP1-cat, 14-3-3 zeta/delta, FAK1, 14-3-3, GSK3 alpha/beta, IGF-1 receptor, Beta-catenin, HSP105, TBLR1, TGIF, AKT(PKB), RNF220, Dsh, Frizzled, Rac1, YAP1 (YAp65), CDK1 (p34)
**10**	Transcription_HIF-1 targets	7.16E-11	G3P2, PDK1, Mxi1, TGF-beta 2, GLUT1, MSH2, ALDOA, Adrenomedullin, HXK2, HXK1, P4HA2, LOXL4, LOXL2, GPI, TGM2, PLAUR (uPAR), P4HA1, Cyclin G2, 5'-NTD, ENO1, LDHA, Alpha-1B adrenergic receptor, HGF receptor (Met), MSH6, PGK1, AK3, PKM2, IBP3
**11**	TGF-beta 1-induced transactivation of membrane receptors signaling in HCC	7.85E-11	alpha-3/beta-1 integrin, E-cadherin, ITGB1, Laminin 5, PI3K cat class IA, ITGA2, alpha-6/beta-1 integrin, Cyclin E, SLUG, FAK1, alpha-2/beta-1 integrin, Beta-catenin, Cyclin A, TGF-beta, Cyclin D1, ITGA6, AKT(PKB), Fibronectin, Rac1, CDK2
**12**	Cell cycle_Chromosome condensation in prometaphase	1.69E-10	Cyclin B, Histone H3, CAP-G, CAP-G/G2, CAP-H/H2, CAP-C, Cyclin A, TOP2, BRRN1, Aurora-A, CAP-E, Histone H1, CDK1 (p34)
**13**	IGF family, invasion and metastasis in colorectal cancer	1.88E-10	RhoA, alpha-3/beta-1 integrin, E-cadherin, ITGB1, PI3K cat class IA, ITGAV, PLAT (TPA), IRS-1, IGF-1 receptor, Beta-catenin, PLAUR (uPAR), alpha-V/beta-6 integrin, Fibronectin, alpha-V/beta-5 integrin, PLAU (UPA), Rac1
**14**	Cell adhesion_PLAU signaling	4.09E-10	Casein kinase II, alpha chains, RhoA, alpha-3/beta-1 integrin, c-IAP2, PI3K cat class IA, LAMC2, Survivin, c-IAP1, alpha-V/beta-1 integrin, EGFR, G-protein alpha-i family, MRLC, FAK1, PLAUR

			(uPAR), CDC42, G-protein alpha-i3, AKT(PKB), sUPAR, alpha-V/beta-5 integrin, PLAU (UPA), Paxillin, Rac1
**15**	Transcription_Role of heterochromatin protein 1 (HP1) family in transcriptional silencing	6.72E-10	TIF1-beta, Mi-2, Histone H3, SUMO-1, Cyclin A2, Cyclin E, E2F1, CDC25A, HDAC1, HP1 beta, E2I, HP1 gamma, HP1, Histone H4, CDK1 (p34), MYOG, MYOD
**16**	DNA damage_ATM/ATR regulation of G2/M checkpoint: nuclear signaling	6.99E-10	WDHD1, CDC18L (CDC6), HSF1, Chk1, Wee1, Cyclin B, Cyclin B2, CDC25C, 14-3-3 sigma, CDH1, Cyclin A, TTK, RBBP8 (CtIP), Cyclin B1, Claspin, PLK1, CDK1 (p34), CDK2
**17**	Beta-catenin-dependent transcription regulation in colorectal cancer	9.59E-10	COX-2 (PTGS2), AKT1, MTS1 (S100A4), LAMC2, LAMC2 (80kDa), CD44, CD44 soluble, Fascin, MMP-14, CD44 (EXT), Beta-catenin, PLAUR (uPAR), Cyclin D1, LAMC2 (100kDa), PLAU (UPA), YAP1 (YAp65)
**18**	HGF signaling in colorectal cancer	1.95E-09	COX-2 (PTGS2), E-cadherin, Laminin 5, PI3K cat class IA, AKT1, LAMC2, CD44, EGFR, FAK1, Beta-catenin, PLAUR (uPAR), AKT2, Cyclin D1, AKT(PKB), LAMB3, HGF receptor (Met), ADAM10, MACC1, LAMA3 (Epiligrin), Rac1, mTOR
**19**	Mechanisms of drug resistance in SCLC	2.03E-09	alpha-3/beta-1 integrin, HSF1, ITGB1, p73, PI3K cat class IA, Survivin, alpha-6/beta-1 integrin, c-IAP1, alpha-V/beta-1 integrin, FAK1, TOP2 alpha, Bcl-XL, alpha-2/beta-1 integrin, IGF-1 receptor, Cyclin D1, AKT(PKB), CD9, HSP70, PDK (PDPK1), Fibronectin, Rad51, mTOR
**20**	Cytoskeleton remodeling_Regulation of actin cytoskeleton organization by the kinase effectors of Rho GTPases	1.19E-08	MLCP (cat), RhoA, PAK, Rac1-related, Cdc42 subfamily, Spectrin, MRLC, PRK1, RhoA-related, Myosin II, Cortactin, CDC42, Alpha-actinin, RhoC, MyHC, Rac3, Paxillin, Rac1, MRCK

**21**	G-protein signaling_RhoA inhibition	1.92E-08	RhoA, Cyclin B, SUMO-1, NET1, SMURF1, 14-3-3 sigma, 14-3-3 zeta/delta, p190RhoGAP, FAK1, RhoE, PKC-zeta, GEF720, CDC42, TGF-beta, RhoGAP5, RacGAP1, E2I, Aurora-A, BDNF, Rac1, CDK1 (p34), Tubulin (in microtubules)
**22**	IGF-1 signaling in multiple myeloma	3.73E-08	RhoA, ITGB1, c-IAP2, PI3K cat class IA, Survivin, IRS-2, IRS-1, FAK1, GSK3 alpha/beta, IGF-1 receptor, CDC42, AKT(PKB), PDK (PDPK1), Fibronectin, Paxillin, mTOR, CDK1 (p34)
**23**	Glucocorticoid-induced elevation of intraocular pressure as glaucoma risk factor	4.06E-08	COX-2 (PTGS2), RhoA, ITGB1, Laminin 5, PI3K cat class IA, PLAT (TPA), PI3K cat class IA (p110-beta), Filamin B (TABP), EGFR, FAK1, Syndecan-4, CDC42, Alpha-actinin, LAMB3, HGF receptor (Met), Fibronectin, alpha-V/beta-5 integrin, CD47, Rac1
**24**	Role of adhesion of SCLC cells in tumor progression	5.61E-08	RhoA, alpha-3/beta-1 integrin, E-cadherin, ITGB1, alpha-6/beta-1 integrin, alpha-V/beta-1 integrin, MMP-14, FAK1, alpha-2/beta-1 integrin, CDC42, PTHrP, CD9, Fibronectin, Rac1
**25**	Cell cycle_Nucleocytoplasmic transport of CDK/Cyclins	7.73E-08	CRM1, Cyclin E, Cyclin D, Importin (karyopherin)-alpha, Cyclin A, Cyclin D1, Cyclin B1, CDK1 (p34), CDK2
**26**	DNA damage_Double-strand break repair via homologous recombination	8.34E-08	WDHD1, Ku80, RAD18, SPIDR, AUNIP, STARING, RAD54B, RAD54L, Histone H2B, MAD2b, RecQL4, RMI2, RBBP8 (CtIP), Rad51, PIR51, PLK1, Histone H2A, Histone H4, HROB, USP4, CDK1 (p34), CDK2
**27**	Development_PIP3 signaling in cardiac myocytes	9.21E-08	PI3K cat class IA, IRS-1, Cyclin D, 14-3-3, GSK3 alpha/beta, PKC-zeta, Bcl-XL, IGF-1 receptor, G-protein beta/gamma, CDC42, AKT(PKB), PDK (PDPK1), HGF receptor (Met), p90Rsk, GYS1, mTOR
**28**	Cell cycle_Influence of Ras and Rho proteins on G1/S Transition	9.93E-08	MLCP (cat), RhoA, PI3K cat class IA, Cyclin A2, Cyclin E, E2F1, MRLC, FAK1, RGL2, CDC42, Cyclin D1, AKT(PKB), PDK (PDPK1), RalA, Rac1, CDK2, CDK6
**29**	Chemoresistance pathways mediated by constitutive	1.12E-07	alpha-3/beta-1 integrin, Laminin 5, PI3K cat class IA, LAMC2, Survivin, alpha-6/beta-1 integrin, IRS-1, FAK1, Bcl-XL, alpha-2/beta-1 integrin, IGF-1 receptor, AKT(PKB), PDK (PDPK1), Fibronectin, mTOR

	activation of PI3K pathway and BCL-2 in small cell lung cancer		
**30**	G-protein signaling_RhoA activation	1.31E-07	RhoA, MPP5, Cyclin B, INADL, NET1, SMURF1, EGFR, G-protein alpha-i family, VAV-2, p190RhoGAP, FAK1, IGF-1 receptor, G-protein beta/gamma, GEF720, TGF-beta, Ephrin-A receptors, Ephrin-A, Reticulon 4, Fibronectin, PLK1, ECT2, CDK1 (p34)
**31**	Cell cycle_Start of DNA replication in early S phase	1.35E-07	CDC18L (CDC6), ORC1L, RPA3, MCM4, ORC4L, Cyclin E, E2F1, MCM10, ORC6L, MCM2, RPA2, Histone H1, CDK2
**32**	Cell adhesion_ECM remodeling	1.83E-07	Laminin 5, MMP-13, CD44, SERPINE2, Caveolin-2, PLAT (TPA), EGFR, Kallikrein 2, MMP-14, MMP-12, IGF-1 receptor, PLAUR (uPAR), Fibronectin, Kallikrein 1, LAMA4, PLAU (UPA), Kallikrein 3 (PSA)
**33**	Leptin signaling in colorectal cancer	2.28E-07	E-cadherin, PI3K cat class IA, Survivin, Cyclin E, FAK1, Bcl-XL, Beta-catenin, CDC42, Cyclin D1, AKT(PKB), Cyclin B1, ADAM17, Rac1, mTOR, CDK1 (p34)
**34**	Development_YAP/TAZ-mediated co-regulation of transcription	2.45E-07	p63, GATA-3, p73, Survivin, RUNX2, RHAMM, KLF5, UFO, SLUG, FOXM1, Bcl-XL, Beta-catenin, HBP17, Cyclin D1, UCA1, YAP1 (YAp65), CDK6
**35**	IGF-1 receptor/EGFR cooperation in lung cancer	2.88E-07	PI3K cat class IA, Survivin, IRS-2, IRS-1, EGFR, PKC-zeta, IGF-1 receptor, AKT(PKB), PDK (PDPK1), p90Rsk, mTOR
**36**	Cell adhesion_Integrin-mediated cell adhesion and migration	3.02E-07	RhoA, alpha-3/beta-1 integrin, ITGB1, Laminin 5, PKC, alpha-6/beta-1 integrin, PKC-lambda/iota, VASP, 14-3-3 zeta/delta, VAV-2, FAK1, PKC-zeta, alpha-2/beta-1 integrin, CDC42, Alpha-actinin, Fibronectin, Paxillin, Rac1
**37**	Mechanisms of resistance to EGFR inhibitors in lung cancer	3.19E-07	E-cadherin, PI3K cat class IA, Survivin, TACSTD2 (TROP2), IRS-1, EGFR, Claudin-4, IGF-1 receptor, Beta-catenin, AKT(PKB), HSP90, PDK (PDPK1), HGF receptor (Met), Fibronectin, mTOR

**38**	Cell cycle_Role of SCF complex in cell cycle regulation	3.25E-07	Chk1, UBE1, Wee1, CKS1, Cyclin E, E2F1, NEDD8, Cyclin D1, CDC25A, PLK1, CDK1 (p34), CDK2
**39**	Cytoskeleton remodeling_Regulation of actin cytoskeleton nucleation and polymerization by Rho GTPases	4.42E-07	RhoA, RhoD, DRF, Profilin, Rac1-related, Cdc42 subfamily, VASP, FMNL3, RhoA-related, CDC42, mDIA2(DIAPH3), RhoC, Rac3, FNBP1L, Rac1
**40**	Development_Regulation of cytoskeleton proteins in oligodendrocyte differentiation and myelination	5.63E-07	MLCP (cat), Tubulin beta, RhoA, Tubulin alpha, alpha-6/beta-1 integrin, MRLC, Tubulin beta 4, p190RhoGAP, FAK1, chTOG, Myosin II, Cortactin, CDC42, MELC, Paxillin, Rac1, Tubulin (in microtubules)
**41**	TGF-beta signaling via SMADs in breast cancer	6.06E-07	E-cadherin, TGF-beta 2, MMP-13, MTS1 (S100A4), RUNX2, CD44, ANGPTL4, SLUG, CD44 soluble, Fascin, MMP-14, ITGB5, PTHrP, HMGA2, IL-11
**42**	Canonical WNT signaling pathway in colorectal cancer	6.49E-07	Galectin-3, E-cadherin, PI3K cat class IA, AKT1, Survivin, CD44, WNT, BMP4, SLUG, DVL-1, DKK1, Beta-catenin, Cyclin D1, ENC1, HGF receptor (Met), Dsh, Frizzled, Rac1
**43**	Possible regulation of HSF-1/ chaperone pathway in Huntington's disease	6.52E-07	HSF1, Calmodulin, GSK3 alpha/beta, HSP90 alpha, HSP90, PLA2, HSP70, E2I, PLK1, p23 co-chaperone
**44**	Cell cycle_Cell cycle (generic schema)	6.52E-07	Cyclin B, CDC25C, Cyclin E, E2F1, Cyclin D, Cyclin A, CDC25A, CDK1 (p34), CDK2, CDK6

**45**	Pro-oncogenic action of Estradiol/ Estrogen receptors in ovarian cancer	7.33E-07	Keratin 4, BETA-IG-H3, Keratin 7, MALL, PI3K cat class IA, Survivin, CD44, Fra-1, Cyclin A2, NCOA3 (pCIP/SRC3), Cyclin E, EGFR, AKT(PKB), Fibronectin, 14-3-3 theta, PLAU (UPA), IBP3
**46**	Signal transduction_Ephrin-B signaling	8.23E-07	RhoA, HGK(MAP4K4), PAK, Ephrin-B receptor 4, NCK1, Ephrin-B, Ephrin-B receptor 2, GRB7, FAK1, CDC42, Ephrin-B receptor 1, Ephrin-B receptor 6, Ephrin-B2, Ephrin-B receptors, Rac1
**47**	Development_Negative regulation of WNT/Beta-catenin signaling in the cytoplasm	8.99E-07	E-cadherin, SIAH1, Porf-2, STK3, WNT, E2F1, PP1-cat, Presenilin 1, Itch, GSK3 alpha/beta, FAF1, Beta-catenin, G-protein beta/gamma, Cyclin D1, Dsh, Frizzled, YAP1/TAZ, Rac1, YAP1 (YAp65), CDK6
**48**	Nicotine / nAChR alpha-7 signaling in NSCLC	1.09E-06	PI3K cat class IA, Calmodulin, PKC-lambda/iota, Cyclin E, E2F1, Cyclin D1, AKT(PKB), AP-2A, Calpain 1(mu), PDK (PDPK1), Fibronectin, p90Rsk, mTOR, CDK2
**49**	The role of PTEN and PI3K signaling in melanoma	1.48E-06	E-cadherin, PI3K cat class IA, AKT1, Survivin, Livin, N-Ras, FAK1, Beta-catenin, AKT2, AKT(PKB), RhoC, PDK (PDPK1), Fibronectin, Rac1, mTOR
**50**	Chemotaxis_Lysophosphatidic acid signaling via GPCRs	1.55E-06	RhoA, E-cadherin, PAK, AKT1, PKC, alpha-6/beta-1 integrin, PI3K cat class IA (p110-beta), EGFR, G-protein alpha-i family, PLC-delta 1, PRK1, FAK1, PKC-zeta, Bcl-XL, Beta-catenin, G-protein beta/gamma, Rho GTPase, CDC42, AKT(PKB), G-protein alpha-q/11, PDK (PDPK1), ADAM17, Paxillin, Rac1, mTOR, YAP1 (YAp65)

Supplementary Table S2: Pathway analysis of genes coexpressed KDM5A from TCGA pancreatic adenocarcinoma databases using the MetaCore database (with p<0.05 set as the cutoff value).

#	Maps	p-value	Network Objects from Active Data
**1**	DNA damage_Double-strand break repair via homologous recombination	1.20E-16	FIGNL1, WDHD1, SAMHD1, RAD18, RIF1, MRE11, DNA polymerase zeta, BRIP1, RYBP, Brca2, MRN complex, SMARCAD1, CCDC98 (Abraxas), ATM, MCM8, RNF168, NEK1, RMI1, SMARCA5, RPA1, TOPBP1, RBBP8 (CtIP), NUCKS, FANCM, PIR51, Nibrin, WRN, RNF20, HEL308, Histone H4, p53BP1
**2**	Immune response_IL-6 signaling pathway via MEK/ERK and PI3K/AKT cascades	1.72E-15	STAT3, JAK2, PI3K reg class IA (p85), gp130, PI3K cat class IA, GSK3 beta, PI3K reg class IA, GAB1, JAK1, K-RAS, SHP-2, N-Ras, XIAP, ERK1/2, CREB1, IL-6, EGR1, SOS, AKT(PKB), sIL6-RA, PDK (PDPK1), ADAM10, p27KIP1, ADAM17, IL-6 receptor, IP3 receptor, mTOR, IL6RA
**3**	DNA damage_Intra S-phase checkpoint	3.85E-15	HBOA, RAD18, MLL1 (HRX), RIF1, Mitotic cohesin complex, Sirtuin1, MAPKAPK2, BRIP1, SMC3, MRN complex, SMC1, PCAF, MCM4, HUWE1, ATM, beta-TrCP, DNA-PK, ASCIZ, DCLRE1B, Rad50, ATR, TOPBP1, Claspin, Nibrin, TLK1, p38 MAPK, FBXW11, p53BP1
**4**	Development_The role of GDNF ligand family/ RET receptor in cell survival, growth and proliferation	1.82E-14	STAT3, p38alpha (MAPK14), GDNF, ROCK, PI3K reg class IA (p85), PI3K cat class IA, B-Raf, NCK1, GAB1, MEKK1(MAP3K1), SHP-2, N-Ras, XIAP, ATF-1, ERK1/2, FAK1, CREB1, EGR1, p90RSK2(RPS6KA3), CRK, SOS, NF-kB, FRS2, AKT(PKB), PDK (PDPK1), ARTN, IL-22, JNK1(MAPK8), IP3 receptor, MEK4(MAP2K4)
**5**	DNA damage_ATR activation by DNA damage	1.95E-13	Sirtuin1, MRN complex, TTI1, HUWE1, USP7, ATM, LARG, ERK1/2, NEK1, Rad50, Rhino, ATR, p300, TOPBP1, RBBP8 (CtIP), FANCM, Claspin, Nibrin, TLK1, SNIP1, p90Rsk, p53BP1
**6**	IL-6 signaling pathway in lung cancer	3.15E-13	STAT3, JAK2, gp130, PI3K cat class IA, PI3K reg class IA, GAB1, JAK1, ATF-2, SHP-2, EGFR, ERK1/2, IL-6, NF-kB, AKT(PKB), PDK (PDPK1), p38 MAPK, IL-6 receptor, IL6RA

**7**	Androgen receptor activation and downstream signaling in Prostate cancer	5.73E-13	STAT3, Androgen receptor, gp130, Rb protein, B-Raf, GAB1, JAK1, K-RAS, c-Cbl, GCR, NCOA3 (pCIP/SRC3), PI3K cat class IA (p110-beta), SHP-2, EGFR, N-Ras, IL-6, NCOA1 (SRC1), SKP2, PAR1, SOS, FRS2, ERK2 (MAPK1), AKT(PKB), PDK (PDPK1), NCOA2 (GRIP1/TIF2), ADAM10, Elk-4, ADAM17, ERK1 (MAPK3), IL-6 receptor, IL6RA
**8**	Regulation and signaling of HGF receptor (Met) and MSP receptor (RON) in lung cancer	7.98E-13	STAT3, HGF, p38alpha (MAPK14), PI3K reg class IA (p85), PI3K cat class IA, GSK3 beta, Rb protein, NCK1, GAB1, K-RAS, c-Cbl, MEKK1(MAP3K1), ATF-2, ERK1/2, FAK1, CREB1, CrkL, IL-6, Beta-catenin, SOS, AKT(PKB), PDK (PDPK1), HGF receptor (Met), p90Rsk, IL-6 receptor
**9**	Development_Negative regulation of WNT/Beta-catenin signaling in the cytoplasm	1.55E-12	WWP1, HECTD1, LATS1, STK4, c-Cbl, VHL, HIPK2, HUWE1, APC protein, Amer1, beta-TrCP, Itch, GSK3 alpha/beta, Beta-catenin, CYLD, WDR26, Skp2/TrCP/FBXW, TAZ, PI3K cat class III (Vps34), SENP2, JNK1(MAPK8), Frizzled, YAP1/TAZ, YAP1 (YAp65), G-protein alpha-13, CDK6
**10**	Development_Stimulation of differentiation of mouse embryonic fibroblasts into adipocytes by extracellular factors	3.18E-12	CBP, PI3K reg class IA (p85), PI3K cat class IA (p110-alpha), HIVEP2, BMP receptor 2, DRIP130, ATF-2, SHP-2, Adenylate cyclase, XIAP, ATF-1, ERK1/2, CREB1, BMPR1A, p90RSK2(RPS6KA3), SMAD1, PKA-reg (cAMP-dependent), SOS, ERK2 (MAPK1), PDK (PDPK1), p38 MAPK, ERK1 (MAPK3), mTOR, LIF receptor
**11**	ERBB family and HGF signaling in gastric cancer	3.18E-12	STAT3, HGF, JAK2, PI3K cat class IA, Amphiregulin, GSK3 beta, PI3K reg class IA, MEKK1(MAP3K1), EGFR, Neuropilin-1, ERK1/2, Beta-catenin, EGR1, SOS, ERK2 (MAPK1), AKT(PKB), PDK (PDPK1), HGF receptor (Met), p27KIP1, p38 MAPK, ERK1 (MAPK3)
**12**	DNA damage_ATM-dependent double-strand break foci	4.36E-12	JMJD2A, CBP, HBXAP, MRN complex, BRM, Mi-2 beta, USP7, CCDC98 (Abraxas), ATM, BMI-1, RNF168, KDM2A, Mi-2 alpha, BAF180, SMARCA5, PIAS1, BAZ1A, p300, Bard1, Nibrin, RNF20, Histone H4, p53BP1

**13**	Ligand-independent activation of Androgen receptor in Prostate Cancer	6.25E-12	STAT3, Androgen receptor, JAK2, PI3K cat class IA, GSK3 beta, B-Raf, PI3K reg class IA, GAB1, K-RAS, NCOA3 (pCIP/SRC3), EGFR, N-Ras, Beta-catenin, PP2A regulatory, NCOA1 (SRC1), SOS, FRS2, ERK2 (MAPK1), AKT(PKB), PDK (PDPK1), NCOA2 (GRIP1/TIF2), ERK1 (MAPK3), Frizzled
**14**	IL-6 signaling in multiple myeloma	8.74E-12	STAT3, JAK2, PI3K reg class IA (p85), gp130, PI3K cat class IA, GSK3 beta, Rb protein, JAK1, K-RAS, SHP-2, N-Ras, ERK1/2, IL-6, SOS, AKT(PKB), PDK (PDPK1), p27KIP1, IL-6 receptor, IL6RA, CDK6
**15**	DNA damage_p53 activation by DNA damage	8.89E-12	p38alpha (MAPK14), CBP, Sirtuin1, USP10, SMG1, TTC5 (Strap), MEKK1(MAP3K1), HIPK2, PCAF, USP7, ATM, DNA-PK, B56G, PP2A regulatory, MARKK, JNK(MAPK8-10), ATR, p300, p38 MAPK, SET7, DYRK2, MEK4(MAP2K4), RFWD3
**16**	Signal transduction_S1P2 receptor activation signaling	9.39E-12	STAT3, MLCP (reg), SMAD5, JAK2, ROCK, PI3K reg class IA (p85), G-protein alpha-12 family, PI3K cat class IA, GSK3 beta, BMP receptor 2, beta-TrCP, LARG, ROCK1, ERK1/2, FAK1, CREB1, SMAD9 (SMAD8), Beta-catenin, EGR1, SMAD1, JNK(MAPK8-10), MSK1, NF-kB, AKT(PKB), PDK (PDPK1), p38 MAPK, IP3 receptor, YAP1 (YAp65), G-protein alpha-13
**17**	EGFR signaling in Prostate Cancer	9.72E-12	STAT3, Androgen receptor, PI3K reg class IA (p85), gp130, PI3K cat class IA, NCOA4 (ARA70), K-RAS, c-Cbl, EGFR, N-Ras, ERK1/2, SOS, ERK2 (MAPK1), AKT(PKB), PDK (PDPK1), NCOA2 (GRIP1/TIF2), p27KIP1, ERK1 (MAPK3), mTOR
**18**	Effect of H. pylori infection on gastric epithelial cell proliferation	2.35E-11	p38alpha (MAPK14), NF-AT4(NFATC3), B-Raf, SHP-2, EGFR, TLR4, ERK1/2, CREB1, CrkL, EGR1, Skp2/TrCP/FBXW, CRK, SKP2, SOS1, HGF receptor (Met), p27KIP1, Calcineurin B (regulatory), NF-AT, Histone H4, IP3 receptor, MEK4(MAP2K4)
**19**	Development_EGFR signaling pathway	2.45E-11	STAT3, p38alpha (MAPK14), JAK2, PI3K reg class IA (p85), p120GAP, PI3K cat class IA, Amphiregulin, GSK3 beta, NCK1, JAK1, c-Cbl, EGFR, ERK1/2, FAK1, SOS, NF-kB, ERK2 (MAPK1), AKT(PKB), PDK (PDPK1), JNK1(MAPK8), p38 MAPK, ERK1 (MAPK3), MEK4(MAP2K4)

**20**	Chemotaxis_Lysophosphatidic acid signaling via GPCRs	5.40E-11	MLCP (reg), ROCK, PI3K reg class IA (p85), G-protein alpha-12 family, GSK3 beta, PAK, PI3K cat class IA (p110-beta), ATF-2, EGFR, TRAF6, LARG, ROCK1, MKL2, ERK1/2, FAK1, CREB1, Beta-catenin, EGR1, N-CoR, TAZ, CRK, JNK(MAPK8-10), MSK1, AKT(PKB), PDK (PDPK1), ADAM17, p38 MAPK, IP3 receptor, mTOR, YAP1 (YAp65), MEK4(MAP2K4)
**21**	Development_Negative regulation of STK3/4 (Hippo) pathway and positive regulation of YAP/TAZ function	6.96E-11	MLCP (reg), G-protein alpha-12 family, LATS1, PARD3, STK4, HIPK2, EGFR, Itch, LARG, ERK1/2, Mol1b, TAZ, JNK(MAPK8-10), PAR1, RhoGAP5, PJA2, PDK (PDPK1), ERK1 (MAPK3), MASK, NEDD4, YAP1 (YAp65)
**22**	IL-6 signaling in Prostate Cancer	7.01E-11	STAT3, Androgen receptor, gp130, PI3K cat class IA, PI3K reg class IA, GAB1, JAK1, SHP-2, IL-6, NCOA1 (SRC1), SOS, ERK2 (MAPK1), AKT(PKB), PDK (PDPK1), p27KIP1, IL-6 receptor, IL6RA
**23**	Transcription_Hypoxia- and receptor-mediated HIF-1 activation	1.01E-10	STAT3, HGF, ARNT, CBP, PI3K cat class IA, GSK3 beta, LRRK2, NCOA3 (pCIP/SRC3), VHL, EGFR, IGF-2, ERK1/2, NCOA1 (SRC1), AKT(PKB), p300, PDK (PDPK1), NCOA2 (GRIP1/TIF2), p38 MAPK
**24**	Immune response_B cell antigen receptor (BCR) pathway	1.09E-10	PI3K reg class IA (p85), GSK3 beta, Rb protein, B-Raf, NCK1, NF-kB1 (p50), K-RAS, c-Rel (NF-kB subunit), MEKK1(MAP3K1), ATF-2, ETS1, MALT1, N-Ras, NF-kB p50/c-Rel, PIP5KIII, ERK1/2, GSK3 alpha/beta, EGR1, NF-kB, AKT(PKB), SOS1, PDK (PDPK1), Calcineurin B (regulatory), p38 MAPK, TAK1(MAP3K7), IP3 receptor, mTOR, CDK6
**25**	Development_Positive regulation of WNT/Beta-catenin signaling in the cytoplasm	1.17E-10	HECTD1, Trabid, BIG1, Tankyrases, BIG2, USP9X, HIPK2, USP25, USP7, APC protein, FAK1, GSK3 alpha/beta, PP2C alpha, Beta-catenin, PKA-reg type II (cAMP-dependent), JNK(MAPK8-10), ERK2 (MAPK1), AKT(PKB), USP47, Frizzled, SET7, ZBED3, YAP1 (YAp65)

**26**	Development_HGF signaling pathway	1.55E-10	STAT3, HGF, PI3K cat class IA, GSK3 beta, PI3K reg class IA, GAB1, MEKK1(MAP3K1), ERK1/2, CrkL, Beta-catenin, EGR1, CRK, JNK(MAPK8-10), SOS, AKT(PKB), PDK (PDPK1), HGF receptor (Met), MEK4(MAP2K4)
**27**	Immune response_Oncostatin M signaling via MAPK	1.87E-10	gp130, OSM receptor, JAK1, MEKK1(MAP3K1), SHP-2, ERK1/2, LIFR, EGR1, JNK(MAPK8-10), SOS, OSMR, ERK2 (MAPK1), p38 MAPK, ERK1 (MAPK3), MEK4(MAP2K4), LIF receptor
**28**	Ovarian cancer (main signaling cascades)	1.92E-10	HGF, Androgen receptor, PI3K cat class IA (p110-alpha), PI3K cat class IA, GSK3 beta, NCOA4 (ARA70), B-Raf, PI3K reg class IA, K-RAS, MEKK1(MAP3K1), EGFR, ERK1/2, CREB1, IL-6, Beta-catenin, PKA-reg (cAMP-dependent), SOS, NF-kB, AKT(PKB), HGF receptor (Met), PI3K cat class IB (p110-gamma)
**29**	HGF receptor (Met) and MSP receptor (RON) signaling pathways in SCLC	2.78E-10	STAT3, HGF, p38alpha (MAPK14), PI3K reg class IA (p85), PI3K cat class IA, Rb protein, MAPKAPK2, GAB1, ERK1/2, FAK1, CREB1, CrkL, JNK(MAPK8-10), AKT(PKB), PDK (PDPK1), HGF receptor (Met), mTOR
**30**	Development_Flt3 signaling	4.27E-10	PI3K reg class IA (p85), PI3K cat class IA, GAB1, c-Cbl, MEKK1(MAP3K1), ATF-2, SHP-2, ERK1/2, CrkL, CRK, SOS, AKT(PKB), PDK (PDPK1), JNK1(MAPK8), p38 MAPK, p90Rsk, MEK4(MAP2K4)
**31**	Signal transduction_RANKL-dependent osteoclast differentiation	4.83E-10	p38alpha (MAPK14), CBP, PI3K cat class IA, GSK3 beta, PI3K reg class IA, NF-kB1 (p50), eIF2AK3, NF-AT1(NFATC2), PCAF, TRAF6, CREB1, JNK(MAPK8-10), NF-kB, TAB2, AKT(PKB), PDK (PDPK1), ATP6V1C1, CaMK IV, JNK1(MAPK8), p38 MAPK, TAK1(MAP3K7), Cathepsin K, CDK6
**32**	Development_Thromboxane A2 signaling pathway	5.11E-10	PI3K reg class IA (p85), G-protein alpha-12 family, PI3K cat class IA, B-Raf, PI3K reg class IA, EGFR, Adenylate cyclase, ERK1/2, GSK3 alpha/beta, CREB1, IL-6, Beta-catenin, MSK1/2 (RPS6KA5/4), PKA-reg (cAMP-dependent), AKT(PKB), p38 MAPK, IP3 receptor, G-protein alpha-13

**33**	Development_Gastrin in cell growth and proliferation	5.36E-10	STAT3, p38alpha (MAPK14), JAK2, PI3K reg class IA (p85), PI3K cat class IA, PI3K reg class IA (p85-alpha), MEKK1(MAP3K1), EGFR, ERK1/2, FAK1, CREB1, Beta-catenin, JNK(MAPK8-10), SOS, ERK2 (MAPK1), PDK (PDPK1), ERK1 (MAPK3), p90Rsk, IP3 receptor, MEK4(MAP2K4)
**34**	Immune response_TLR2 and TLR4 signaling pathways	6.70E-10	TLR1, PI3K reg class IA (p85), PI3K cat class IA, NF-kB1 (p105), TLR6, TLR4, TRAF6, ERK1/2, CREB1, IL-6, MSK1/2 (RPS6KA5/4), JNK(MAPK8-10), NF-kB, TAB2, AKT(PKB), PDK (PDPK1), p38 MAPK, TAK1(MAP3K7), p90Rsk, TAB3, MEK4(MAP2K4)
**35**	Apoptosis and survival_NGF/ TrkA PI3K-mediated signaling	1.01E-09	TRIO, MLCP (reg), ROCK, PI3K reg class IA (p85), PI3K cat class IA, GSK3 beta, PARD3, ELMO2, GAB1, APC protein, PAK2, ERK1/2, p190RhoGAP, KIDINS220, CREB1, SOS, SSH2L, AKT(PKB), PDK (PDPK1), Calcineurin B (regulatory), SSH1L, mTOR
**36**	Signal transduction_IGF-1 receptor signaling pathway	1.08E-09	STAT3, Androgen receptor, JAK2, PI3K reg class IA (p85), PI3K cat class IA, GSK3 beta, GAB1, JAK1, SHP-2, IGF-2, ERK1/2, FAK1, SOS, AKT(PKB), PDK (PDPK1), JNK1(MAPK8), Bim, mTOR, MEK4(MAP2K4)
**37**	TGF-beta signaling via kinase cascades in breast cancer	1.08E-09	p38alpha (MAPK14), Amphiregulin, ITGAV, NF-kB1 (p50), ATF-2, TGF-beta receptor type II, EGFR, XIAP, TRAF6, ERK1/2, FAK1, JNK(MAPK8-10), TAB2, AKT(PKB), SOS1, ADAM17, p38 MAPK, TAK1(MAP3K7), TAB3
**38**	Immune response_CD40 signaling in B cells	1.38E-09	STAT3, c-IAP2, PI3K reg class IA (p85), PI3K cat class IA, MAPKAPK2, TRAF3, NF-kB1 (p50), c-Cbl, c-Rel (NF-kB subunit), MEKK1(MAP3K1), c-IAP1, TRAF6, ERK1/2, IL-6, JNK(MAPK8-10), NF-kB, AKT(PKB), Dynein 1, cytoplasmic, intermediate chains, p27KIP1, p38 MAPK, Bim, SPAG9, MEK4(MAP2K4)
**39**	Cell cycle_Influence of Ras and Rho proteins on G1/S Transition	1.53E-09	STAT3, MLCP (reg), PI3K cat class IA, GSK3 beta, Rb protein, PI3K reg class IA, ROCK2, ATF-2, ERK1/2, FAK1, RGL2, SKP2, AKT(PKB), PDK (PDPK1), p27KIP1, JNK1(MAPK8), MEK4(MAP2K4), CDK6

**40**	EGFR signaling pathway in lung cancer	1.53E-09	STAT3, HGF, JAK2, PI3K reg class IA (p85), gp130, PI3K cat class IA, Amphiregulin, EGFR, ERK1/2, IL-6, EGR1, SOS, NF-kB, AKT(PKB), PDK (PDPK1), HGF receptor (Met), ADAM17, mTOR
**41**	Development_VEGF signaling via VEGFR2 - generic cascades	1.73E-09	p120GAP, PI3K cat class IA, GSK3 beta, MAPKAPK2, NCK1, PI3K reg class IA, MEKK1(MAP3K1), PAK2, ROCK1, ERK1/2, FAK1, CREB1, Neurofibromin, Beta-catenin, MSK1, SOS, ERK2 (MAPK1), AKT(PKB), PDK (PDPK1), Calcineurin B (regulatory), p38 MAPK, ERK1 (MAPK3), p90Rsk, IP3 receptor
**42**	Development_Androgen receptor in non-reproductive systems development	2.25E-09	Androgen receptor, AdipoR2, GASC1, CBP, ROCK, PI3K cat class IA, BAF155, GSK3 beta, PI3K reg class IA (p85-alpha), Brca2, NCOA3 (pCIP/SRC3), PCAF, EGFR, ERK1/2, Beta-catenin, NCOA1 (SRC1), SOS, AKT(PKB), JMJD1A, p300, PDK (PDPK1), NCOA2 (GRIP1/TIF2)
**43**	Immune response_Gastrin in inflammatory response	3.34E-09	p38alpha (MAPK14), PI3K reg class IA (p85), PI3K cat class IA, MEKK1(MAP3K1), ATF-2, EGFR, TRAF6, LARG, FAK1, CREB1, JNK(MAPK8-10), SOS, MEF2, ERK2 (MAPK1), AKT(PKB), PDK (PDPK1), ERK1 (MAPK3), TAK1(MAP3K7), IP3 receptor, MEK4(MAP2K4)
**44**	Deficient alpha-MSH signaling in melanoma	3.54E-09	p38alpha (MAPK14), PI3K cat class IA, GSK3 beta, Rb protein, B-Raf, PI3K reg class IA, N-Ras, ROCK1, ERK1/2, CREB1, PKA-reg (cAMP-dependent), AKT(PKB), PDK (PDPK1), p38 MAPK
**45**	Development_Estrogen-independent activation of ESR1 and ESR2	3.98E-09	CBP, PI3K cat class IA (p110-alpha), PI3K cat class IA, PI3K reg class IA, NCOA3 (pCIP/SRC3), EGFR, ERK1/2, PKA-reg (cAMP-dependent), NCOA1 (SRC1), SOS, ERK2 (MAPK1), AKT(PKB), p300, PDK (PDPK1), NCOA2 (GRIP1/TIF2), ERK1 (MAPK3)
**46**	Influence of smoking on activation of EGFR signaling in lung cancer cells	3.98E-09	p38alpha (MAPK14), Amphiregulin, B-Raf, MEKK1(MAP3K1), EGFR, Adenylate cyclase, ATF-1, ERK1/2, CREB1, PKA-reg (cAMP-dependent), JNK(MAPK8-10), SOS, ERK2 (MAPK1), ADAM17, ERK1 (MAPK3), MEK4(MAP2K4)
**47**	HGF signaling in melanoma	4.97E-09	HGF, PI3K reg class IA (p85), PI3K cat class IA, GSK3 beta, B-Raf, GAB1, ATF-2, N-Ras, ERK1/2, Beta-catenin, EGR1, JNK(MAPK8-10), AKT(PKB), HGF receptor (Met), p38 MAPK

**48**	Cell cycle progression in Prostate Cancer	4.97E-09	STAT3, Androgen receptor, JAK2, GSK3 beta, Rb protein, ERK1/2, IL-6, Beta-catenin, AKT(PKB), p27KIP1, p38 MAPK, IL-6 receptor, Frizzled, mTOR, CDK6
**49**	Immune response_TNF-R2 signaling pathways	5.83E-09	c-IAP2, PI3K reg class IA (p85), PI3K cat class IA, TRAF3, PI3K reg class IA, c-IAP1, SMURF2, ERK1/2, JNK(MAPK8-10), NF-kB, AKT(PKB), PDK (PDPK1), ZFP91, p38 MAPK, Bim, MEK4(MAP2K4)
**50**	DNA damage_Nucleotide excision repair	5.84E-09	OTUD4, Arkadia, Dicer, Usp24, DNA polymerase kappa, RAD23B, USP7, Ash1, NEDD8, ERCC6, RFC1, XPC, POLR2A, XPF, ATR, p300, SUPT16H, NEDD4, UBXD7, Histone H4

Supplementary Table S3: Pathway analysis of genes coexpressed KDM5B from TCGA pancreatic adenocarcinoma databases using the MetaCore database (with p<0.05 set as the cutoff value).

#	Maps	p-value	Network Objects from Active Data
**1**	Development_Positive regulation of WNT/Beta-catenin signaling in the cytoplasm	2.67E-17	Casein kinase II, alpha chains, COX-2 (PTGS2), GSKIP, HECTD1, ITGB1, Bcl-9, LRP5/LRP6, BIG1, Tankyrases, BIG2, SIAH1, IRS-1, USP25, Tcf(Lef), WNT, 14-3-3 zeta/delta, Alpha-1 catenin, FAK1, 14-3-3, TGT, GSK3 alpha/beta, PP2C alpha, Beta-catenin, HSP105, TBLR1, JNK(MAPK8-10), TGIF, Dsh, USP47, Frizzled, Rac1, YAP1 (YAp65)
**2**	HGF signaling in colorectal cancer	6.47E-13	COX-2 (PTGS2), E-cadherin, Laminin 5, Shc, PI3K cat class IA, GSK3 beta, LAMC2, PTEN, EGFR, XIAP, TCF7L2 (TCF4), ERK1/2, FAK1, Beta-catenin, PLAUR (uPAR), VEGF-A, Cyclin D1, JNK(MAPK8-10), LAMB3, HGF receptor (Met), ADAM10, c-Src, MACC1, LAMA3 (Epiligrin), Rac1
**3**	Development_Negative regulation of WNT/Beta-catenin signaling in the cytoplasm	9.44E-13	KLHL12, WWP1, E-cadherin, HECTD1, LRP5/LRP6, WNT5A, SIAH1, STK3, Tcf(Lef), WNT, HUWE1, Presenilin 1, Itch, Alpha-1 catenin, GSK3 alpha/beta, Casein kinase I epsilon, Beta-catenin, WDR26, Cyclin D1, SENP2, Dsh, JNK1(MAPK8), Casein kinase I alpha, Frizzled, YAP1/TAZ, Rac1, YAP1 (YAp65), CDK6
**4**	TGF-beta 1-induced transactivation of membrane receptors signaling in HCC	8.70E-12	alpha-3/beta-1 integrin, E-cadherin, ITGB1, Laminin 5, PI3K cat class IA, GSK3 beta, ITGA2, alpha-6/beta-1 integrin, PTEN, DOCK1, FAK1, alpha-2/beta-1 integrin, Beta-catenin, TGF-beta, Cyclin D1, ITGA6, PAK1, Fibronectin, c-Src, Rac1, CDK2
**5**	IGF family, invasion and metastasis in colorectal cancer	1.46E-11	alpha-3/beta-1 integrin, E-cadherin, ITGB1, PI3K cat class IA, ITGAV, PLAT (TPA), IRS-1, Alpha-1 catenin, ITGB4, Beta-catenin, PLAUR (uPAR), alpha-V/beta-6 integrin, alpha-6/beta-4 integrin, Fibronectin, alpha-V/beta-5 integrin, ERK1 (MAPK3), Rac1
**6**	Effect of H. pylori infection on gastric epithelial cells motility	1.74E-11	E-cadherin, ITGB1, MARK2, Vinculin, ZO-1, DOCK1, ERK1/2, FAK1, Occludin, Beta-catenin, Cortactin, Alpha-actinin, JNK(MAPK8-10), PAK1, JAM1, p120-catenin, c-Src, Alpha-catenin, Rac1

**7**	Development_Positive regulation of WNT/Beta-catenin signaling in the nucleus	3.45E-11	Casein kinase II, alpha chains, SMYD2, SOX11, LRP5/LRP6, GSK3 beta, UCHL5, FHL2, Tcf(Lef), RUNX, WNT, XIAP, Alpha-1 catenin, TCF7L2 (TCF4), Beta-catenin, TBLR1, SOX4, BCL9/B9L, NCOA2 (GRIP1/TIF2), Dsh, TDG, Casein kinase I alpha, Frizzled, UBR5, YAP1 (YAp65)
**8**	Development_VEGF signaling via VEGFR2 - generic cascades	4.19E-11	COX-2 (PTGS2), Shc, p120GAP, PI3K cat class IA, GSK3 beta, MAPKAPK2, Calcineurin A (catalytic), Calmodulin, PKC, NCK1, Vinculin, PAK2, TCF7L2 (TCF4), ERK1/2, FAK1, CREB1, Neurofibromin, Beta-catenin, PLAUR (uPAR), VEGF-A, Fyn, PAK1, HSP90, c-Src, Calcineurin B (regulatory), ERK1 (MAPK3), Rac1, IP3 receptor
**9**	Proliferative action of Gastrin in gastric cancer	2.78E-10	E-cadherin, Shc, PI3K cat class IA, PKC, IRS-1, EGFR, TCF7L2 (TCF4), ERK1/2, SP3, FAK1, CREB1, Beta-catenin, G-protein alpha-q, Cyclin D1, PAK1, G-protein alpha-q/11, SP1, ICRF, c-Src, IP3 receptor
**10**	Inhibition of Ephrin receptors in colorectal cancer	4.10E-10	E-cadherin, ROCK, c-Rel (NF-kB subunit), KLF5, Ephrin-B, WNT, TCF7L2 (TCF4), FAK1, Beta-catenin, Ephrin-A receptors, Ephrin-A, Ephrin-B2, Ephrin-A receptor 2, Frizzled, Rac1
**11**	Cell adhesion_Integrin-mediated cell adhesion and migration	1.44E-09	alpha-3/beta-1 integrin, ITGB1, Laminin 5, PARD3, PKC, alpha-6/beta-1 integrin, Vinculin, PKC-lambda/iota, PINCH, VASP, 14-3-3 zeta/delta, DOCK1, LARG, FAK1, alpha-2/beta-1 integrin, Alpha-actinin, PAK1, Fibronectin, c-Src, PARD6, Rac1
**12**	CHDI_DEGs from Replication data_Causal network	1.48E-09	Shc, GSK3 beta, Calcineurin A (catalytic), Calmodulin, ADAM9, PKC, PTEN, cPLA2, WNT, TCF7L2 (TCF4), ERK1/2, FAK1, LRP5, CREB1, Casein kinase I epsilon, alpha-2/beta-1 integrin, Beta-catenin, CtBP, G-protein alpha-q, SP1, Calcineurin B (regulatory), Dsh, Frizzled, IP3 receptor
**13**	Development_WNT/Beta-catenin signaling pathway. Signalosome	2.63E-09	E-cadherin, LRP5/LRP6, GSK3 beta, WNT, LRP6, GSK3 alpha/beta, Casein kinase I epsilon, G-protein alpha-q, PAK1, ARF1, PIP5KI, p120-catenin, PPP2R5E, Dsh, Casein kinase I alpha, Caprin-2, Frizzled

**14**	Cytoskeleton remodeling_Integrin outside-in signaling	3.63E-09	TRIO, alpha-3/beta-1 integrin, alpha-11/beta-1 integrin, GSK3 beta, Vinculin, PINCH, Tcf(Lef), ERK1/2, FAK1, alpha-2/beta-1 integrin, Beta-catenin, Cyclin D1, Alpha-actinin, PAK1, Fibronectin, c-Src, Rac1, WIRE
**15**	Cell adhesion_Tight junctions	4.00E-09	ROCK, Cingulin, PARD3, PDZ-RhoGEF, MPP5, PKC-lambda/iota, INADL, AF-6, N-WASP, ZO-1, MRLC, Occludin, Cortactin, JAM1, PARD6, DNMBP(TUBA), ZO-2
**16**	Development_WNT/Beta-catenin signaling in the cytoplasm	4.41E-09	Casein kinase II, alpha chains, E-cadherin, LRP5/LRP6, GSK3 beta, Tcf(Lef), WNT, MACF1, IPMK, GSK3 alpha/beta, Casein kinase I epsilon, Beta-catenin, G-protein alpha-q, PAK1, p120-catenin, c-Src, Dsh, Casein kinase I alpha, Frizzled, Rac1
**17**	Signal transduction_S1P1 receptor signaling	4.67E-09	COX-2 (PTGS2), Shc, PI3K cat class IA (p110-alpha), GSK3 beta, RelA (p65 NF-kB subunit), MAPKAPK2, Calmodulin, PI3K cat class IA (p110-beta), KLF5, Tcf(Lef), cPLA2, EGFR, ZO-1, ERK1/2, FAK1, PI3K class II (CII-alpha), Beta-catenin, Cortactin, VEGF-A, Fyn, Cyclin D1, Alpha-actinin, JNK(MAPK8-10), PAK1, c-Src, Alpha-catenin, Rac1, IP3 receptor, YAP1 (YAp65)
**18**	G-protein signaling_RhoA activation	5.62E-09	TRIO, MALS-3, Cingulin, PDZ-RhoGEF, MPP5, ARHGEF2, INADL, NET1, SMURF1, EGFR, ZO-1, XIAP, LARG, FAK1, TGF-beta, G-protein alpha-q, Ephrin-A receptors, Ephexin, Ephrin-A, Fibronectin, c-Src, JNK1(MAPK8), ECT2, PTPN12
**19**	Mechanisms of resistance to EGFR inhibitors in lung cancer	6.00E-09	E-cadherin, Shc, Ep-CAM, PI3K cat class IA, ErbB2, K-RAS, PTEN, IRS-1, EGFR, Claudin-4, ErbB3, Beta-catenin, HSP90, HGF receptor (Met), Fibronectin, c-Src, ERK1 (MAPK3)
**20**	Glucocorticoid-induced elevation of intraocular pressure as glaucoma risk factor	6.75E-09	TRIO, COX-2 (PTGS2), ITGB1, Laminin 5, ROCK, PI3K cat class IA, WNT5A, PLAT (TPA), PI3K cat class IA (p110-beta), Filamin B (TABP), EGFR, FAK1, Alpha-actinin, PAK1, LAMB3, HGF receptor (Met), Fibronectin, c-Src, alpha-V/beta-5 integrin, Rac1

**21**	Cytoskeleton remodeling_FAK signaling	8.71E-09	Shc, PI3K cat class IA, GSK3 beta, Calmodulin, TRAF3, PTEN, DOCK1, ERK1/2, FAK1, alpha-2/beta-1 integrin, VEGF-A, PAK1, G-protein alpha-q/11, Fibronectin, c-Src, Calpain 2(m), JNK1(MAPK8), Rac1, IP3 receptor
**22**	E-cadherin signaling and its regulation in gastric cancer	9.72E-09	E-cadherin, Formin, Shc, GSK3 beta, WNT, EGFR, Beta-catenin, Alpha-actinin, IQGAP1, HGF receptor (Met), p120-catenin, c-Src, Dsh, Alpha-catenin, Frizzled
**23**	Cell cycle_Influence of Ras and Rho proteins on G1/S Transition	1.53E-08	PI3K cat class IA, GSK3 beta, RelA (p65 NF-kB subunit), ROCK2, ATF-2, DIA1, Tob1, MRLC, ERK1/2, FAK1, RGL2, Cyclin D1, PAK1, RalA, JNK1(MAPK8), Rac1, CDK2, CDK6
**24**	Signal transduction_Ephrin-B signaling	1.88E-08	ROCK, HGK(MAP4K4), p120GAP, PAK, NCK1, AF-6, Ephrin-B, N-WASP, GRB7, ERK1/2, FAK1, JNK(MAPK8-10), PAK1, c-Src, Ephrin-B2, NUMB, Rac1
**25**	Development_EGFR signaling via small GTPases	2.30E-08	Shc, p120GAP, PI3K cat class IA, Rab-5A, EPS8, ErbB2, K-RAS, EGFR, N-Ras, RNTRE, ERK1/2, JNK(MAPK8-10), E3b1(ABI-1), Rac1
**26**	Signal transduction_Ephrin-A signaling	2.98E-08	Shc, ROCK, Ephrin-A1, NCK1, N-WASP, ERK1/2, CREB1, Ephrin-A5, Ephrin-A4, Ephrin-A receptors, Ephexin, Ephrin-A, c-Src, Ephrin-A receptor 4, Ephrin-B2, Ephrin-A receptor 2, Rac1, Ephrin-A2
**27**	Leptin signaling in colorectal cancer	3.19E-08	E-cadherin, PI3K cat class IA, GSK3 beta, RelA (p65 NF-kB subunit), PTEN, TCF7L2 (TCF4), ERK1/2, FAK1, Beta-catenin, VEGF-A, Cyclin D1, JNK(MAPK8-10), Leptin receptor, c-Src, ADAM17, Rac1
**28**	Development_Androgen receptor in non-reproductive systems development	3.25E-08	AdipoR2, Shc, ROCK, PI3K cat class IA, BAF155, GSK3 beta, GRP78, DSPP, NCOA3 (pCIP/SRC3), Tcf(Lef), EGFR, ERK1/2, HSPA1B, Beta-catenin, VEGF-A, JMJD1A, HSP70, NCOA2 (GRIP1/TIF2), c-Src, NUMB, HSPA1A, IBP3
**29**	Chemotaxis_Lysophosphatidic acid signaling via GPCRs	3.28E-08	E-cadherin, ROCK, GSK3 beta, PAK, PDZ-RhoGEF, PLC-eta 1, PKC, alpha-6/beta-1 integrin, Vinculin, PI3K cat class IA (p110-beta), ATF-2, DIA1, Tcf(Lef), EGFR, TRAF6, LARG, ERK1/2, PRK1, FAK1, CREB1, Beta-catenin, Rho GTPase, JNK(MAPK8-10), G-protein alpha-q/11, c-Src, ADAM17, Rac1, IP3 receptor, YAP1 (YAp65)

**30**	Mitogenic action of ErbB2 in breast cancer	4.09E-08	Shc, PI3K cat class IA, GSK3 beta, BRK, ErbB2, EGFR, TCF7L2 (TCF4), ERK1/2, ErbB3, Beta-catenin, Cyclin G2, Cyclin D1, NF-kB, PAK1, ER81, c-Src, NUMB, Rac1
**31**	Development_Negative regulation of STK3/4 (Hippo) pathway and positive regulation of YAP/TAZ function	4.13E-08	PARD3, PDZ-RhoGEF, ARHGEF2, STK3, ASPP1, EGFR, Itch, ASPP2, LARG, ERK1/2, Mol1b, JNK(MAPK8-10), RASSF6, RhoGAP5, G-protein alpha-q/11, ERK1 (MAPK3), MASK, YAP1 (YAp65), ZO-2
**32**	Development_WNT/Beta-catenin signaling in the nucleus	4.13E-08	LRP5/LRP6, TNIK, BAF53, Tcf(Lef), RUNX, WNT, TCF7L2 (TCF4), Beta-catenin, MED12, PYGO2, JNK(MAPK8-10), SMARCA5, BCL9/B9L, MEF2, NIPBL, p120-catenin, Dsh, Frizzled, UBR5
**33**	Role of alpha-6/beta-4 integrins in carcinoma progression	4.62E-08	Plectin 1, Laminin 5, PI3K cat class IA, ErbB2, IRS-1, EGFR, ErbB3, ITGB4, 14-3-3 gamma, VEGF-A, alpha-6/beta-4 integrin, MSP receptor (RON), PAK1, HGF receptor (Met), Rac1, IP3 receptor
**34**	Cell adhesion_Histamine H1 receptor signaling in the interruption of cell barrier integrity	4.62E-08	Histamine H1 receptor, E-cadherin, ROCK, Calmodulin, Vinculin, ZO-1, MRLC, FAK1, Occludin, Beta-catenin, Alpha-actinin, G-protein alpha-q/11, p120-catenin, c-Src, Alpha-catenin, IP3 receptor
**35**	Development_Negative regulation of WNT/Beta-catenin signaling in the nucleus	5.68E-08	NF-AT5, KDM2, LRP5/LRP6, WNT5A, GSK3 beta, Calcineurin A (catalytic), BACH1, Tcf(Lef), WNT, Alpha-1 catenin, TCF7L2 (TCF4), 14-3-3, Casein kinase I epsilon, Beta-catenin, CtBP, VEGF-A, TBLR1, BCL9/B9L, SENP2, Dsh, PPAR-gamma, Frizzled, Histone H1

**36**	Role of Tissue factor in cancer independent of coagulation protease signaling	5.69E-08	alpha-3/beta-1 integrin, Laminin 5, Shc, PI3K cat class IA (p110-alpha), alpha-6/beta-1 integrin, ATF-2, ERK1/2, FAK1, YES, VEGF-A, PAK1, c-Src, ERK1 (MAPK3), Rac1
**37**	Neurophysiological process_Receptor-mediated axon growth repulsion	6.61E-08	ROCK, PDZ-RhoGEF, ROCK2, EGFR, GRB7, LARG, Fer, Cortactin, Fyn, Ephrin-A receptors, Ephexin, PAK1, Ephrin-A, c-Src, Ephrin-A receptor 2, Rac1
**38**	Mechanisms of drug resistance in SCLC	6.79E-08	alpha-3/beta-1 integrin, ABCC1, ITGB1, PI3K cat class IA, GSK3 beta, RelA (p65 NF-kB subunit), alpha-6/beta-1 integrin, alpha-V/beta-1 integrin, XIAP, ERK1/2, FAK1, TOP2 alpha, alpha-2/beta-1 integrin, VEGF-A, Cyclin D1, MGF, HSP70, Caspase-8, Fibronectin, HSPA1A
**39**	Cell adhesion_Endothelial cell contacts by junctional mechanisms	7.52E-08	Plakophilin 4, Cingulin, AF-6, ZO-1, Desmoplakin, Occludin, Beta-catenin, Alpha-actinin, JAM1, p120-catenin, Alpha-catenin, ZO-2
**40**	Cell adhesion_Classical cadherin-mediated cell adhesion	7.52E-08	TRIO, E-cadherin, Formin, Vinculin, N-WASP, Beta-catenin, Cortactin, Alpha-actinin, p120-catenin, PDZ-GEF1, Alpha-catenin, Rac1
**41**	TGF-beta signaling via kinase cascades in breast cancer	7.55E-08	COX-2 (PTGS2), TGF-beta 2, ITGB1, Shc, RelA (p65 NF-kB subunit), ITGAV, ATF-2, EGFR, XIAP, TRAF6, ERK1/2, FAK1, ErbB3, JNK(MAPK8-10), TGF-alpha, c-Src, ADAM17, Rac1
**42**	Cell adhesion_PLAU signaling	9.65E-08	Casein kinase II, alpha chains, alpha-3/beta-1 integrin, Shc, ROCK, PI3K cat class IA, LAMC2, alpha-V/beta-1 integrin, EGFR, XIAP, MRLC, ERK1/2, FAK1, PLAUR (uPAR), NF-kB, PAK1, c-Src, sUPAR, alpha-V/beta-5 integrin, Rac1
**43**	G-protein signaling_RhoA inhibition	9.95E-08	14-3-3 beta/alpha, p120GAP, MARK2, ARHGEF2, SUMO-1, NET1, SMURF1, 14-3-3 zeta/delta, FAK1, 14-3-3 gamma, TGF-beta, Fyn, RhoGAP5, AMPK alpha 1 subunit, PAK1, c-Src, PARD6, ERK1 (MAPK3), SLK, Alpha-catenin, Rac1

**44**	Development_Negative feedback regulation of WNT/Beta-catenin signaling	1.30E-07	KLHL12, LRP5/LRP6, Tcf(Lef), WNT, HUWE1, TCF7L2 (TCF4), GSK3 alpha/beta, Casein kinase I epsilon, Beta-catenin, G-protein alpha-q, Cyclin D1, c-Src, Dsh, Frizzled
**45**	ERBB family and HGF signaling in gastric cancer	1.37E-07	COX-2 (PTGS2), Shc, PI3K cat class IA, GSK3 beta, ErbB2, EGFR, GRB7, TCF7L2 (TCF4), ERK1/2, ErbB3, Beta-catenin, VEGF-A, Cyclin D1, TGF-alpha, SP1, HGF receptor (Met), ERK1 (MAPK3)
**46**	CHDI_Correlations from Discovery data_Causal network	1.46E-07	Shc, PI3K cat class IA, GSK3 beta, Calmodulin, PTEN, WNT, TCF7L2 (TCF4), ERK1/2, TSC-22, CREB1, Beta-catenin, G-protein alpha-q, MEF2, SP1, Calpain 1(mu), c-Src, YY1, Dsh, Frizzled, IP3 receptor
**47**	Stem cells_Aberrant Wnt signaling in medulloblastoma stem cells	1.69E-07	Casein kinase II, alpha chains, GSK3 beta, WNT, TCF7L2 (TCF4), LRP5, Casein kinase I epsilon, Beta-catenin, Cyclin D1, Dsh, Casein kinase I alpha, Frizzled
**48**	G-protein signaling_Rac1 activation	1.87E-07	TRIO, PI3K cat class IA, PAK, EPS8, Ephrin-A1, ErbB2, Rac1-related, K-RAS, AF-6, EGFR, Tiam2, DOCK1, Rho GTPase, DOCK7, ALS2, p200RhoGAP, c-Src, Ephrin-A receptor 4, E3b1(ABI-1), Rac1
**49**	Signal transduction_Ephrin reverse signaling	2.07E-07	ROCK, Ephrin-B, MRLC, DOCK1, Fyn, Ephrin-A receptors, JNK(MAPK8-10), PAK1, Ephrin-A, SFK, c-Src, Rac1
**50**	Development_Gastrin in cell growth and proliferation	2.35E-07	E-cadherin, Shc, PI3K cat class IA, IRS-1, EGFR, TCF7L2 (TCF4), ERK1/2, FAK1, CREB1, Beta-catenin, G-protein alpha-q, Cyclin D1, JNK(MAPK8-10), PAK1, G-protein alpha-q/11, c-Src, ERK1 (MAPK3), IP3 receptor
